# On the influence of the source of porcine colostrum in the development of early immune ontogeny in piglets

**DOI:** 10.1038/s41598-022-20082-1

**Published:** 2022-09-17

**Authors:** Shaiana Salete Maciag, Franciana Volpato Bellaver, Gabrielly Bombassaro, Vanessa Haach, Marcos Antônio Zanella Morés, Lana Flávia Baron, Arlei Coldebella, Ana Paula Bastos

**Affiliations:** 1 Universidade Estadual do Centro-Oeste do Paraná – Campus CEDETEG, Guarapuava, PR Brazil; 2 Instituto Federal Catarinense – Campus Concórdia, Concórdia, SC Brazil; 3 Universidade Federal Do Rio Grande Do Sul, Porto Alegre, RS Brazil; 4 Embrapa Suínos E Aves, Concórdia, SC Brazil

**Keywords:** Cell proliferation, Lymphokines, Thymus, Immunology, Adaptive immunity, Lymphoid tissues

## Abstract

The effects on the ontogeny of serum cytokines and immune cells caused by feeding suckling piglets with sow/gilt colostrum and milk replacer was assessed in the present study. After farrowing, the piglets born were randomized into six groups: GG and SS (n = 10/group): piglets were kept with their dam; GS (n = 10): piglets were changed from gilts to sows; SG (n = 10): piglets were changed from sows to gilts; GMR (n = 6) and SMR (n = 8): piglets from either gilts or sows were isolated from the dams and were bottle-fed ad libitum with commercial formula milk replacer. The piglets remained in the groups during the first 24 h of life and were later returned to their respective mothers. Serum immunoglobulin concentration and lymphocyte proliferation from the blood, spleen, thymus, and mesenteric lymph node of the piglets were assessed at 24 h and at 28 days of age. Serum cytokine concentrations were measured through a cytokine multiplex assay at 24 h. Overall, piglets suckling on sows (SS and GS) had a higher concentration of serum immunoglobulin at 24 h, which was also associated with a rise in plasma cytokine concentration and greater ability of B and T cells from lymphatic organs and blood mononuclear cells to respond to mitogens. We suggest a bias towards Th1-, Th2-, and Th17-cell polarizing and cytokines during the suckling period, which may be influenced by maternal immunological factors in the colostrum, such as dam parity. All findings suggest sow parity having a possible role, which may contribute to exerting a modulating action on immune response development.

## Introduction

The early development of the immune system of pigs occurs almost entirely during gestation. The immune system has minimal activity until birth. This phase, the fetus is protected from antigenic stimuli from pathogenic organisms, with which it has no contact, due to the diffuse epitheliochorial function of the pig’s placenta. Therefore, during gestation, the piglet remains in a sterile, impermeable, and protective environment offered by the uterus, which leads to an absence of antibody production in the fetus^1^. Moreover, the epitheliochorial nature of the pig’s placenta does not allow the transference of maternal immunoglobulin (Igs) to the offspring^2^. Hence, these piglets have agammaglobulinemia at birth and are immunodeficient until weaning^1,3^. After birth, the neonate piglet goes from an intrauterine sterile environment to an external environment that is rich in antigens and pathogenic agents; thus demanding an adequate immune response to survive^4^.

The beginning of immune system activity may be triggered by events during labor, by the colostrum, and by the environment, seeking to promote protection against foreign molecules and microorganisms that are beginning their colonization and challenge the animal^5–8^. In fact, a piglet’s first four weeks of life represent a critical period in which these animals are more susceptible to diseases^9,10^. Many factors contribute to this situation, among which are the immaturity of the newborn piglet’s immune system^1,3,4^. These piglets are not able to develop a satisfactory immune response, since their immune system is still functionally immature and lack the necessary time to generate humoral and cellular immunity^1,11^. Therefore, the components of the immune system are not completely functional in a newborn piglet, requiring a few weeks to reach maturity^2^.

The immune system originates still during the intrauterine phase and, in pigs, hematopoiesis occurs in three different places^12–14^. Initially the first lymphoid organ is the yolk sac, where stem cells emerge, following to the fetal liver, where they are produced^13,14^. Subsequently, they follow to the thymus for the process of cell differentiation, maturation, and selection of T lymphocytes occurs in the thymus cortex and at approximately 40 days of gestation they can be detected in the thymus, receiving the name of double negative thymocytes^15,16^. The process of negative and positive selection of thymocytes is not clear and can happen in either the second or third trimester of gestation, given that during this period there is an intense death of thymocytes^17^. In general terms, a positive selection allows the survival of T cells that express T-cell receptor (TCR) that can recognize the major histocompatibility complex (MHC) of the cells themselves, while a negative selection eliminates cells that connect strongly to their own MHC, an important factor to determine self-tolerance^17–19^.

After birth, bone marrow takes on the role of producing lymphocytes and the thymus begins its involution^20^. During the first weeks of life, the immune system progressives rapidly under the shield of passive maternal immunity that originates from the colostrum^1^. Primary (bone marrow and thymus) and secondary (such as spleen, Peyer’s patches, and lymph nodes) lymphoid organs are the main components of the immune system and their development and maturation carry out an important role in this process^14^.

The immunoglobulin absorption period, which originates from the colostrum, happens during the first hours after the piglet is born and can extend to up to 36 h after labor. Absorption occurs through the intestine by means of macromolecule endocytosis after ingesting the colostrum^21^. The colostrum contains a series of components that act directly and indirectly in the immunological function of the piglet^22^. Immunoglobulins are the most common of these colostrum components and offer direct protection against antigens^23^. The most abundant types in the colostrum are immunoglobulin G (IgG), immunoglobulin A (IgA), and immunoglobulin M (IgM), respectively. After being absorbed by the enterocytes they head to the intestinal lymphatic system, IgA is later sent to mucosa surfaces, while IgG remains in circulation^24^. The concentration of IgG in colostrum is several times greater than in the sow’s plasma and decreases exponentially over the first 24 h after labor^4^.

The colostrum also contains leukocyte cells, such as granulocytes (neutrophils, 40%), T lymphocytes (30%), B lymphocytes (13–16%), and macrophages (7–11%)^25,26^. Pro-inflammatory cytokines IL-1β, IL-6, and TNF-α and anti-inflammatory cytokines such as IL-10 are also present in the colostrum; these substances have an immunostimulant effect on the immune response to mitogens^27^. In piglets either fed with milk replacers and/or substitutes or those that were deprived of sow colostrum, passive immunity transference from sow to litter does not happen and these piglets do not absorb macromolecules, such that the intestinal cells responsible for this absorption represent open vacuoles, demonstrating that there was no macromolecule endocytosis^28^. The present study aimed to evaluate the influence of colostrum from gilts and sows, and colostrum substitutes on the development of the porcine immune system. This was assessed by determining cytokines in the piglets’ serum, cell numbers, function, and phenotypic expression of immune cell surface of isolated lymphocytes from the blood, spleen, mesenteric lymph node, and thymus and their change with age in the neonatal piglet.

## Results

### Clinical evaluation

The average weight of piglets at birth was 1.40 kg, with no significant differences among the groups (Supplementary Table S1). The animals that were fed with milk replacer (GMR and SMR) presented diarrhea around 20–36 h after birth. Eight piglets (five from GMR and three from SMR) died during the experiment, before completing 28 days. The deaths in piglets of the GMR and SMR groups occurred in the first 5–20 days of life and body weight differences and reduced mobility were observed, compared to littermates. In addition, the post-mortem analysis of the piglets did not show any respiratory nor gastrointestinal clinical macroscopic signs. Data from the first 24-h of these animals were excluded from the experiment. No significant lesions were observed in the necropsy of these pigs, but body weight measurements demonstrated that the piglets were not growing normally.

No significant differences were observed among the groups regarding body weight gain during the suckling period at 24 h of age (Supplementary Table S1); but the groups that were fed with milk replacer (GMR and SMR) had a lower body weight gain during the weaning period, at 28 days of age.

### Measurement of immunoglobulin concentrations

**Figure 1:**
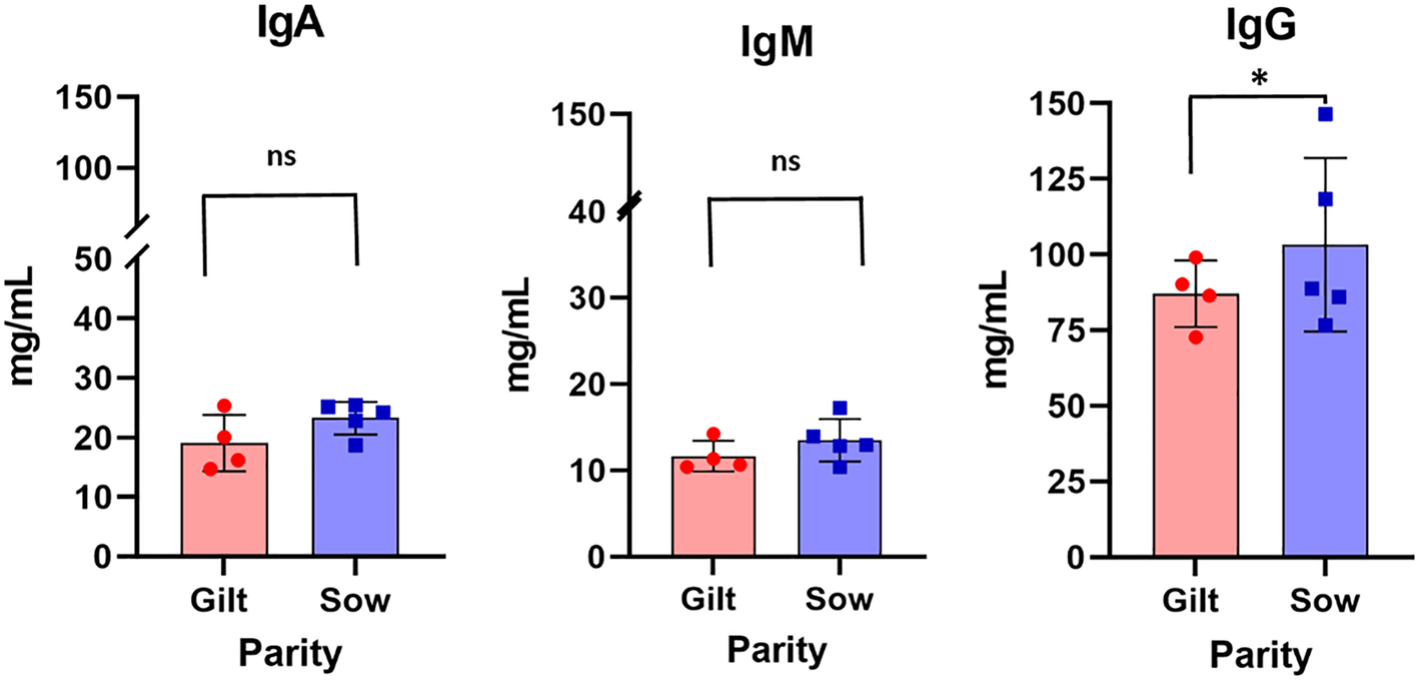
Comparison of total IgG, IgM, and IgA concentrations (mg/mL) in colostrum from gilts and sows in the first 3 h after farrowing. Error bars represent the standard error of the mean (± SEM). Gilt IgA (19.06 ± 1.37) vs. Sow IgA (23.25 ± 1.22), *P* < 0.2207; Gilt IgM (11.67 ± 0.89) vs. Sow IgM (13.49 ± 1.10), *P* < 0.3272; *Gilt IgG (87.09 ± 5.49) vs. Sow IgG (103.27 ± 12.8), *P* < 0.0112.

As expected, sow parity had a significant (*P* < 0.001) effect on colostral IgG concentration, such that the IgG concentrations in the colostrum were higher in sows than in gilts. The IgA and IgM concentrations did not differ between gilts and sows (Fig. 1).

**Figure 2:**
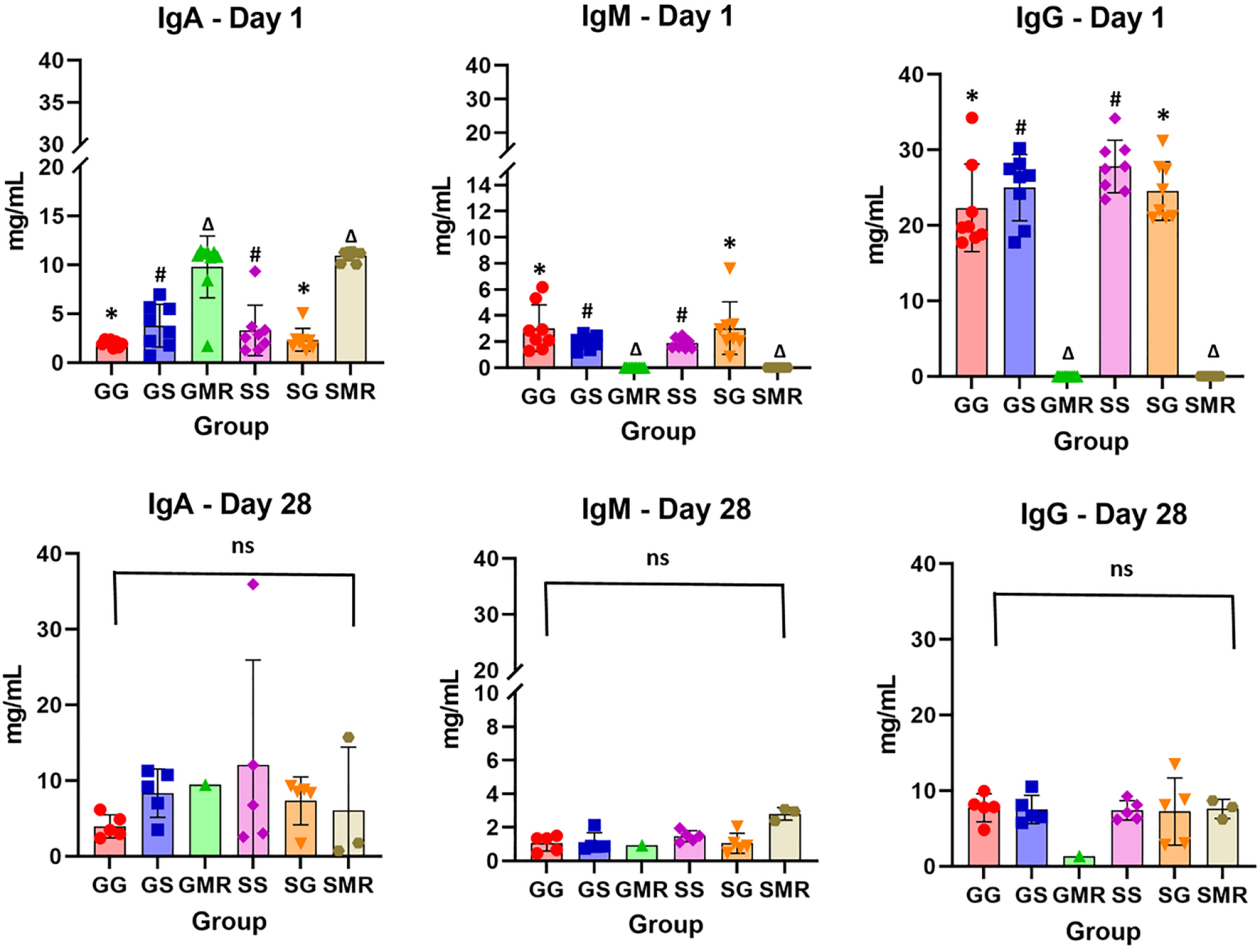
Effect of sow parity and their colostrum on piglet serum immunoglobulin concentration (mg/mL) at 24 h and 28 days after birth. Error bars represent the standard error of the mean (± SEM). Asterisks indicate significant differences among groups (*P* ≤ 0.05).

Piglet serum Ig concentrations at days 1 and 28 of age are indicated in Fig. 2. The IgG and IgM concentration, 24 h from the start of parturition, were consistently lower in piglets fed with milk replacer (GMR, *P* < 0.0001; and SMR, *P* < 0.0001) than in the other groups. Serum IgM concentration was higher in piglets that suckled gilt colostrum (GG 3.025 ± 0.632 mg/mL and SG 3.025 ± 0.712 mg/mL) than in piglets that suckled sow colostrum (GS 1.976 ± 0.177 mg/mL and SS 1.876 ± 0.142 mg/mL). The serum IgG level was higher in the SS group, with a mean value of 27.81 ± 1.23 mg/mL. Interestingly, piglets that suckled sow colostrum, such as the SS (27.81 ± 1.23 mg/mL) and GS (25.00 ± 1.55 mg/mL) groups, had higher serum IgG concentrations than piglets that suckled gilt colostrum, which were represented by the GG (22.32 ± 2.05 mg/mL) and SG (23.54 ± 1.37 mg/mL) groups.

The remaining IgA concentration was also greater in piglets that suckled sow colostrum (3.294 ± 0.714 mg/mL for the SS group and 3.782 ± 0.572 mg/mL for the GS group) when compared to the GG (1.983 ± 0.119 mg/mL) and SG (2.326 ± 0.411 mg/mL) groups, whose piglets suckled gilt colostrum. During lactation at the 28-day mark, the IgA concentration increased three-fold in the SS group (from 3.294 to 12.06 mg/mL) (Fig. 2). The amount of IgA in piglets that suckled sow colostrum and then gilt milk (GS group) was slightly higher than the group that remained with the gilt (GG), however, it was not significantly higher at 28 days (8.346 mg/mL). The animals that received milk replacer (GMR and SMR) were the only ones that did not show an increase in IgA concentration along with lactation.

As expected, IgG, IgM, and IgA concentrations decreased with time in all groups, the levels of these Igs were the highest in the first hour of lactation (Fig. 2). Even so, IgG concentrations in early life were significantly influenced by the parity of the sow providing the colostrum. Piglets that suckled sow colostrum, which were those in the SS and GS groups, showed higher values than those in the GG and SG groups, but this difference did not remain between the groups with mature milk in the weaning period (28 days). Piglets that were not fed with colostrum in the first hours of life but fed with milk replacer (GMR and SMR) showed an increased IgG level on the 28th day of life. IgM concentrations increased over time in the SMR group (from 0 to 2.802 mg/mL, *P* = 0.05) and in the GMR group (from 0 to 0.923 mg/mL, not significant), and showed a tendency for higher concentrations than the other groups.

### Cytokine concentrations in plasma

All cytokines (GM-CSF, IFNγ, IL-1α, IL-1ra, IL-1β, IL-2, IL-4, IL-6, IL-8, IL-10, IL-12, IL-18, and TNFα) evaluated using Luminex were expressed, and the levels of pro- and anti-inflammatory cytokines analyzed among the piglet groups at 24 h of age are shown in Fig. 4 and Supplementary Table S4. To identify if maternal immunological factors transferred to offspring via colostrum play a potential role in ontogeny changes and immunomodulate the immune repertoire of the offspring, the concentration of plasma cytokines and chemokines in dam and piglet groups over the first day of life were measured.

**Figure 3:**
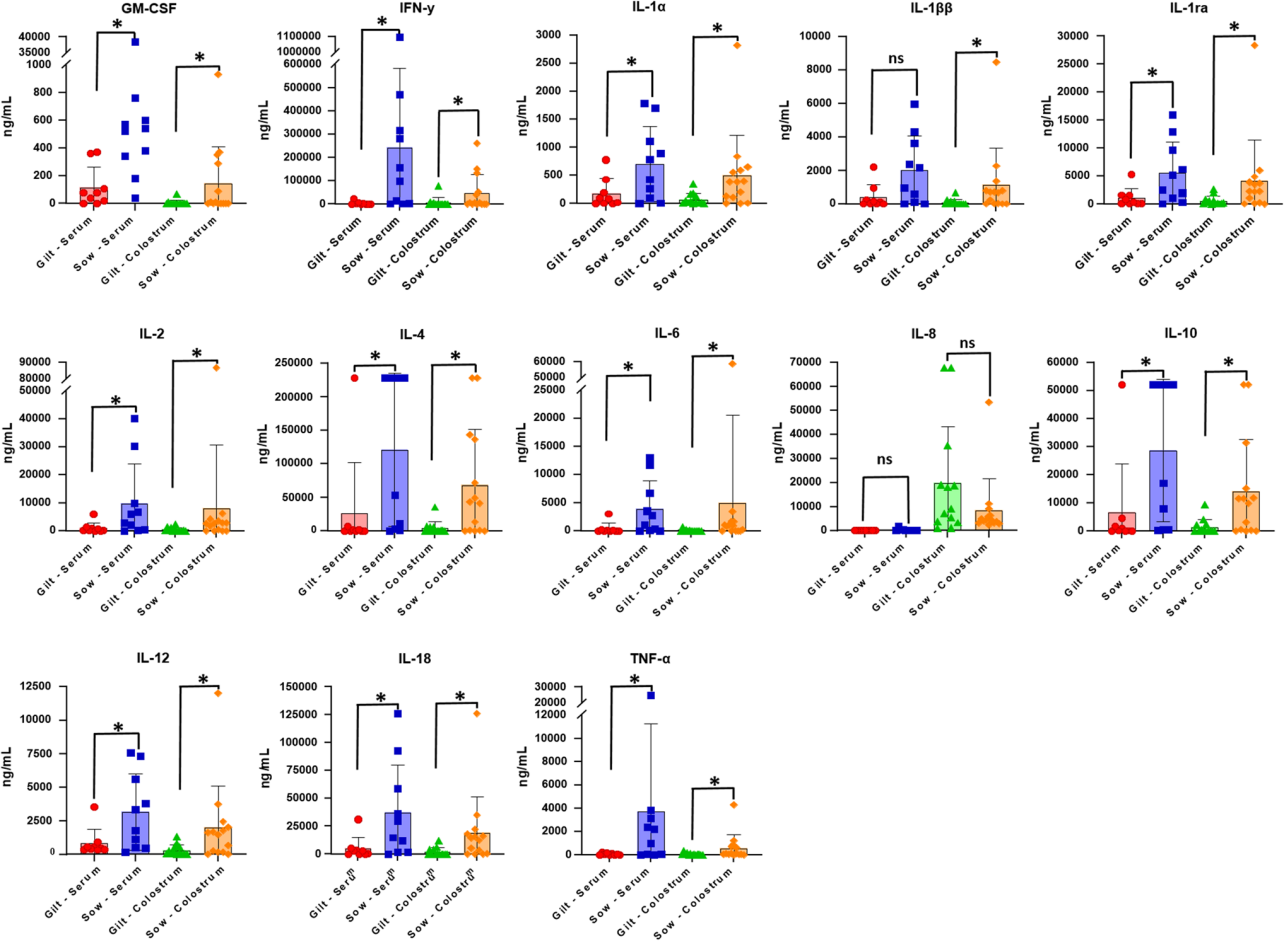
Comparison of cytokine concentrations (ng/mL) in colostrum and serum from gilts and sows in the first 3 h after farrowing. Error bars represent the standard error of the mean (± SEM). Different superscripts indicate significant differences among groups (*P* ≤ 0.05).

Comparing the two groups of dams, GM-CSF, IFNγ, IL-1α, IL-1RA, IL-2, IL-4, IL-6, IL-10, IL-12, IL-18, and TNFα concentrations were significantly higher in sow colostrum (*P* < 0.05) and serum (*P* < 0.05) than in gilts (Fig. 3, Supplementary Tables S2 and S3).

**Figure 4:**
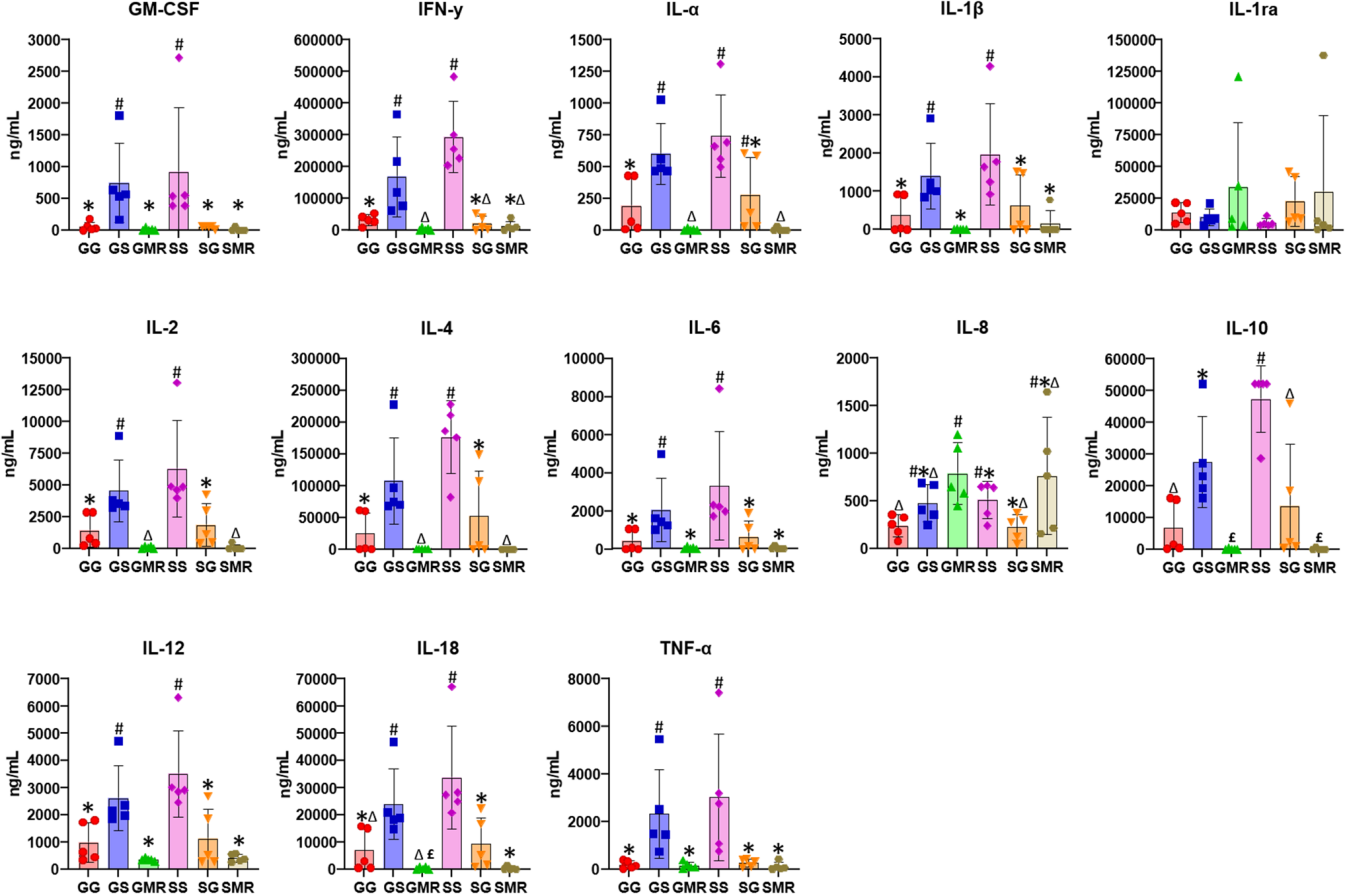
Effect of sow parity and their colostrum on piglet serum cytokine concentration (ng/mL) at 24 h after birth. Error bars represent the standard error of the mean (± SEM). Different superscripts indicate significant differences among groups (*P* ≤ 0.05).

Similar to the analysis of sow cytokines, the concentrations of GM-CSF, IFNγ, IL-1α, IL-1β, IL-2, IL-4, IL-6, IL-10, IL-12, IL-18, and TNFα were significantly higher in the groups with piglets suckling on sow colostrum (SS and GS) than in the groups with gilt colostrum (GG and SG; *P* < 0.05). Finally, the lowest concentrations of these cytokines were observed in the groups of suckling piglets that were fed milk replacer (Fig. 4 and Supplementary Table S4). Overall, we detected a similar developmental pattern for all Th1, Th2, and Th17 cytokine correlates. Nevertheless, the production of IL-1RA and IL-8 was higher in groups of suckling piglets that were fed milk replacer (GMR and SMR) than among piglets that suckled either sow or gilt colostrum (SS, SG, GG, and GS), but these values were not significantly higher (Fig. 4).

### Mitogen-stimulated lymphocytes of various organs

Trypan blue exclusion staining and a microscopic evaluation of viability were conducted right after the primary culture cell (thymus, peripheral blood mononuclear cells-PBMCs, spleen and mesenteric lymph node-mLN) at the pre-determined time points (24 h and 28 days), following a flow cytometric analysis. The analysis by flow cytometry showed that the number of macrophage cells, T and B lymphocytes, increased with the age of the piglets in all lymphatic tissues evaluated.

The gate was performed as described in a previous study conducted by our research group^26^. In summary, the gate was based on forward scatter (FSC) and side scatter (SSC) properties to estimate lymphocyte population and debris exclusion, whereas doublet cells were subjected to doublet plotting considering the forward scatter height (FSC-H) against the forward scatter area (FSC-A). Dead cell discrimination was excluded using 7-AAD staining. Then, according to CD3, CD4, CD8α, CD79a, CD19, CD27, CD25, and macrophage expressions, seven different cell subsets were defined: (i) CD3e^+^CD4^+^, (ii) CD3e^+^CD4^+^CD27^+^, (iii) CD3e^+^CD4^+^CD25^+^, (iv) CD3e^+^CD8α^+^, (v) CD3e^+^CD8α^+^CD27^+^, (vi) CD19^+^CD79a^+^, and (vii) macrophages^+^. Therefore, CD3^+^ cells were considered to represent 100% of T lymphocytes; CD27^+^ represents central memory T cells; CD25^+^ represents regulatory T cells; CD79a^+^ represents B lymphocytes; and macrophages^+^ represent monocyte/macrophage lineages. The foremost predominant immune cell type in lymphatic tissues evaluated were T lymphocytes (Supplementary Table S6).

**Figure 5:**
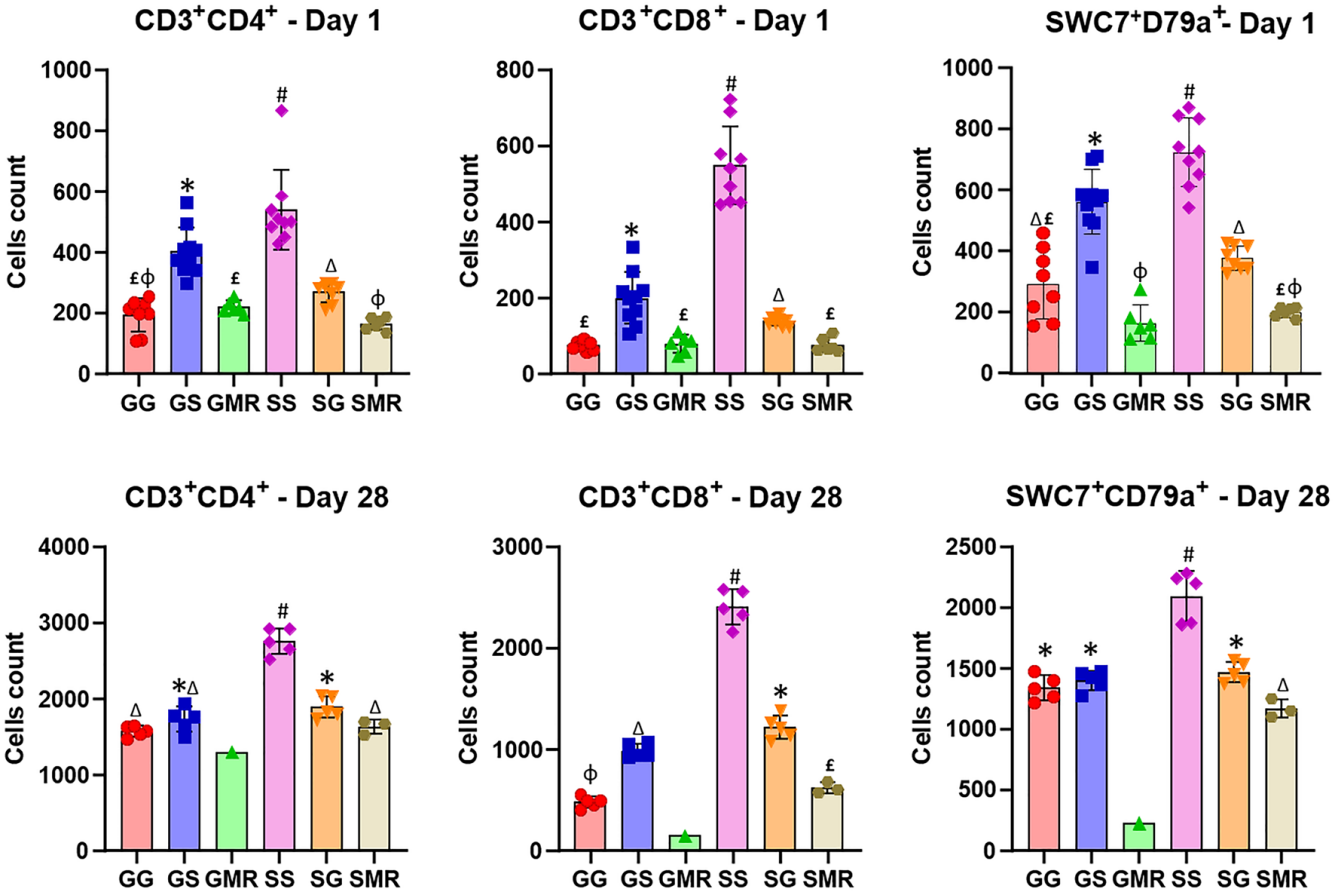
Comparative analysis of CFSE-based lymphocyte proliferation from PBMC in the groups studied. Peripheral blood mononuclear cells were stained with CFSE (1 × 10^6^/mL) and cultured in duplicate. The cells were incubated in a medium alone (nonstimulated-control) and with Concanavalin A (5 µg/mL, stimulated cells). The cells from piglet groups were cultured for 72 h.

PBMCs cells were stained with CFSE and then activated with Concanavalin A for 24 h and 28 days. The flow cytometry analysis results of these cells are given in Fig. 5. Counterstaining with CD3/CD79a and 7-AAD allowed us to gate on live B and T cells and the mean fluorescence intensity of cells. A higher count of activated B (CD19^+^CD79a^+^) and T cells (CD3e^+^CD4^+^ and CD3e^+^CD8α^+^) was observed in groups with piglets that suckled on sow colostrum (SS and GS) at the 24-h mark (Fig. 5 and Supplementary Table S5). A variety of activated cell changes was observed in the weaning period (28 days, Fig. 5 and Supplementary Table S5). The changes from colostrum to mature milk also modified the amount of activated B and T cells in groups of piglets that suckled colostrum from either another dam (GS and SG) or from their dam (GG and SS), and those that were fed a milk replacer (GMR and SMR). In this case, the offspring from sows (SS) that suckled sow’s colostrum and mature milk (after 24 h of life) showed a significantly higher amount of activated T and B cells than their siblings from other groups (SG and SMR), as well as piglets from gilts (GG, GS, and GMR), though this was significantly higher in the SS group (Fig. 5).

A flow cytometry analysis was carried out to determine the colostrum’s potential in activating CD3e^+^CD4^+^ helper T cells, CD3e^+^CD8a^+^ cytotoxic T cells, and T cell subsets in lymphatic organs under Concanavalin A stimulation for 24 h and 28 days of life. The T cell population was more numerous than the B cell population. Spontaneous proliferation by nonstimulated lymphocytes and proliferation of mitogen-stimulated lymphocytes was significantly affected by age, considering lymphocytes isolated from peripheral blood, spleen, thymus, and mesenteric lymph node (Supplementary Table S6). However, piglets fed with milk replacer (GMR and SMR groups) showed lower proliferation lymphocytes than groups fed colostrum (SS, SG, GG, and GS).

**Figure 6:**
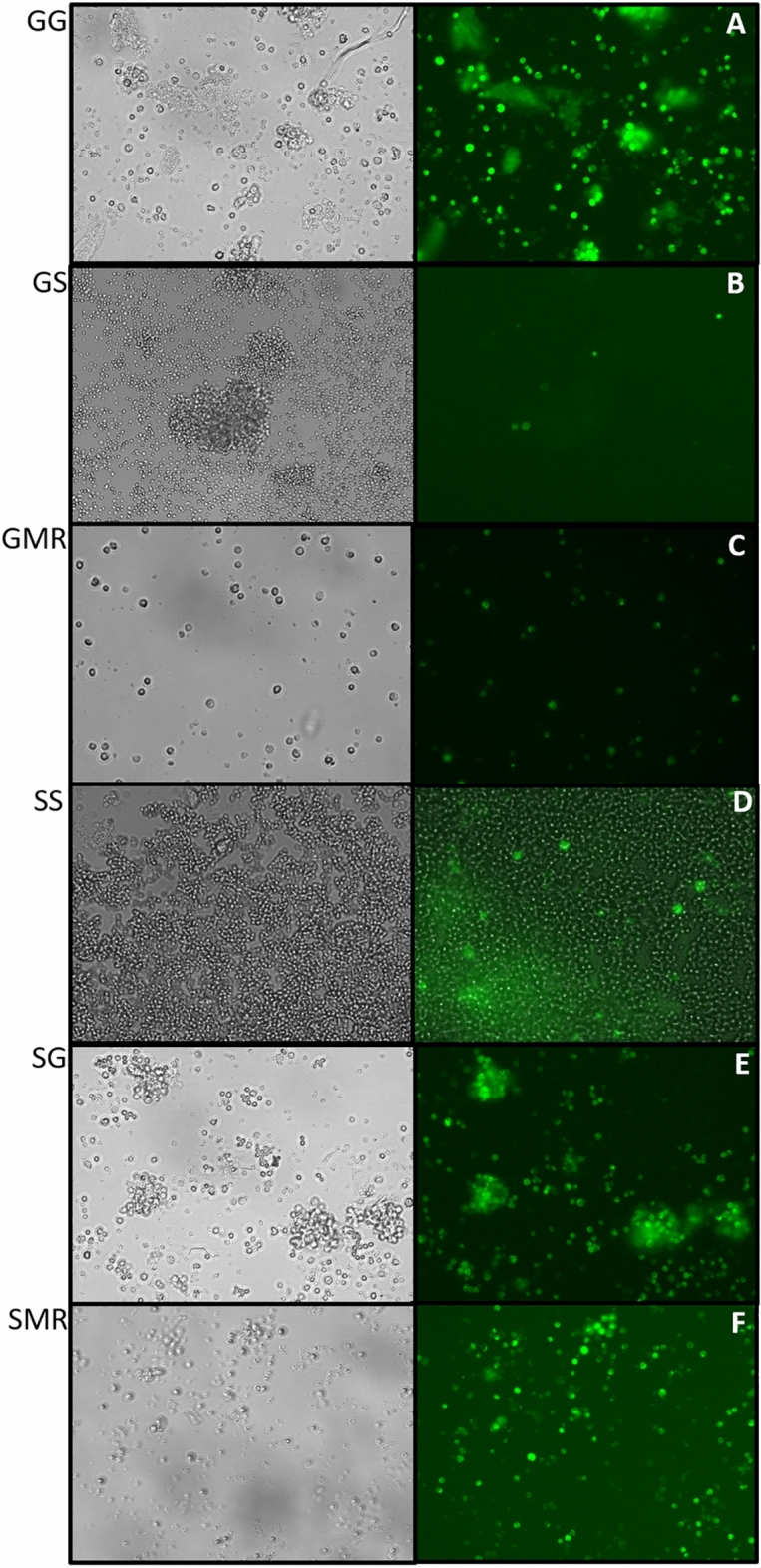
Analysis of CFSE-based lymphocyte proliferation. Thymus cells were stained with CFSE (1 × 10^6^/mL) and cultured in duplicate. The cells were incubated in a medium alone (nonstimulated-control) and with Concanavalin A (5 µg/mL, stimulated cells). The cells from piglet groups were cultured for 96 h. Cells were imaged on an EVOS M7000 microscope at 40X magnification; the same laser and gain settings were used to capture images at each point: (**A**) GG group, (**B**) GS group, (**C**) GMR group, (**D**) SS group, (**E**) SG group, and (**F**) SMR group, all cells were stimulated with Concanavalin A.

In addition, the proliferation of isolated lymphocytes was analyzed using the CFSE dye and presented through the stimulation proliferation index. Interestingly, the proliferation of mitogen-stimulated lymphocytes, when labeled with CFSE, enabled a more thorough analysis and comparison of the differences in lymphocyte proliferation between the groups of piglets (Fig. 6). According to CFSE intensity and number of cells, lymphocyte proliferation was higher in piglets nursed by sows. Subsequently, lymphoid cell subpopulations were also characterized by specific antibodies through flow cytometry.

The sow colostrum increased the CD3e^+^CD4^+^ helper T-cell population and CD4^+^ T cell subsets at the 24-h time point, such as CD4^+^CD25^+^ regulatory T cell and CD3e^+^CD4^+^CD27^+^ naive and/or central memory CD4^+^ T-cell in thymus (CD3e^+^CD4^+^  = *P* < 0.0002; CD3e^+^CD4^+^CD25^+^  = *P* < 0.0001; and CD3e^+^CD4^+^CD27^+^  = *P* < 0.0001) and spleen (CD3e^+^CD4^+^  = *P* < 0.0017; CD3e^+^CD4^+^CD25^+^  = *P* < 0.0091; and CD3e^+^CD4^+^CD27^+^  = *P* < 0.0036), thus these cells populations were significantly influenced by colostrum according to sow parity (Table 1). The SS group showed an increased count of the CD4^+^ population when compared to their littermates in the SMR and SG groups (SS ˃ SG ˃ SMR groups) (*P* < 0.05). Piglets of the GS group also presented a higher content of CD4^+^ population (CD3e^+^CD4^+^, CD3^+^CD4^+^CD25^+^ and CD3e^+^CD4^+^CD27^+^; *P* ≤ 0.05) in comparison to their littermates from the GG and GMR groups.

**Table 1:** Stimulation proliferation index for lymphocytes from lymphoid organs at 24 h of age.

Lymphatic organ	Immune cells	Groups-24 h							Pr > χ^2^
			GG	GS	GMR	SS	SG	SMR	
Spleen	B cells	SWC7^+^CD79a^+^	2029 ± 260^b^	2425 ± 190^b^	1549 ± 387^b^	2860 ± 196^a^	2040 ± 169^b^	1609 ± 338^b^	0.0574
	T cells	CD3e^+^CD4^+^	2447 ± 134^b^	3544 ± 133^ac^	1904 ± 210^b^	3897 ± 228^a^	2641 ± 82^d^	2362 ± 425^bcd^	0.0017
		CD3e^+^CD4^+^CD25^+^	254.00 ± 18.1^bc^	266.60 ± 15.8^b^	197.20 ± 9.3^c^	294.80 ± 52.4^ab^	396.80 ± 38.9^a^	172.00 ± 25.4^c^	0.0091
		CD3e^+^CD4^+^CD27^+^	162.80 ± 11.5^b^	231.20 ± 28.4^a^	142.20 ± 12.0^b^	280.60 ± 30.4^a^	206.80 ± 3.95^a^	121.80 ± 42.4^b^	0.0036
		CD3e^+^CD8α^+^	808.20 ± 220^b^	478.20 ± 23.2^c^	238.60 ± 19.9^c^	1453 ± 226^a^	378.40 ± 74.8^c^	471.00 ± 56.3^c^	0.0009
		CD3e^+^CD8α^+^CD27^+^	84.20 ± 9.83^b^	60.80 ± 11.9^bc^	40.80 ± 3.79^c^	175.40 ± 49.1^a^	151.40 ± 20.1^a^	99.60 ± 10.2^ab^	0.0005
	Myeloid cells	Macrophage^+^	941.40 ± 51.6^a^	770.80 ± 30.3^b^	443.00 ± 53.6^c^	890.00 ± 34.5^a^	759.20 ± 84.1^ab^	531.00 ± 61.8^c^	0.0007
Thymus	B cells	SWC7^+^CD79a^+^	300.20 ± 35.2^c^	442.00 ± 54.1^b^	125.60 ± 10.6^d^	699.60 ± 18.2^a^	386.60 ± 13.9^bc^	103.60 ± 7.66^d^	0.0001
	T cells	CD3e^+^CD4^+^	403.40 ± 19.9^b^	876.40 ± 57.1^c^	401.60 ± 35.8^b^	1150 ± 50.0^a^	527.00 ± 22.6^d^	374.00 ± 18.5^b^	0.0002
		CD3e^+^CD4^+^CD25^+^	123.20 ± 12.6^b^	194.00 ± 4.74^c^	75.80 ± 9.37^d^	347.00 ± 22.6^a^	158.20 ± 4.14^e^	51.00 ± 1.79f.	< .0001
		CD3e^+^CD4^+^CD27^+^	117.00 ± 4.11^b^	679.00 ± 18.2^c^	47.00 ± 4.15^d^	993.00 ± 58.6^a^	258.40 ± 22.5^e^	24.80 ± 1.46f.	< .0001
		CD3e^+^CD8α^+^	549.00 ± 32.9^b^	435.40 ± 49^c^	219.00 ± 18.7^d^	1339 ± 178^a^	682.20 ± 59.6^b^	283.40 ± 3.85^c^	0.0002
		CD3e^+^CD8α^+^CD27^+^	301.20 ± 5.87^b^	230.20 ± 6.31^c^	148.20 ± 37.3^ cd^	674.00 ± 18.0^a^	236.80 ± 9.54^c^	86.40 ± 21.2^d^	< .0001
	Myeloid cells	Macrophage^+^	109.00 ± 9.77^b^	389.00 ± 26.3^c^	56.80 ± 2.58^d^	319.60 ± 22.0^a^	251.40 ± 12.0^e^	72.40 ± 4.40f.	< .0001
mLN	B cells	SWC7^+^CD79a^+^	1047 ± 45^bd^	996.00 ± 57.3^ cd^	840.00 ± 46.5^c^	2740 ± 182^a^	1464 ± 64^e^	1273 ± 106^be^	0.0002
	T cells	CD3e^+^CD4^+^	2372 ± 174^bc^	2058 ± 132^b^	2166 ± 345^b^	4011 ± 350^a^	2971 ± 164^c^	2250 ± 418^b^	0.0062
		CD3e^+^CD4^+^CD25^+^	315.60 ± 20.0^b^	340.00 ± 24.0^b^	299.60 ± 54.5^b^	846.20 ± 33.4^a^	461.80 ± 40.4^c^	484.00 ± 35.2^c^	0.0007
		CD3e^+^CD4^+^CD27^+^	224.80 ± 19.3^bc^	230.20 ± 25.5^b^	158.60 ± 22.6^c^	375.60 ± 27.9^a^	197.60 ± 12.8^bc^	162.60 ± 19.6^c^	0.0031
		CD3e^+^CD8α^+^	2084 ± 24^a^	1583 ± 114^b^	532.20 ± 39.4^c^	2121 ± 120^a^	1585 ± 61^b^	1364 ± 149^b^	0.0002
		CD3e^+^CD8α^+^CD27^+^	419.60 ± 42.7^abc^	362.00 ± 47.1^bc^	258.20 ± 71.4^bc^	653.80 ± 96.7^a^	592.20 ± 53.4^a^	282.40 ± 34.6^c^	0.0056
	Myeloid cells	Macrophage^+^	181.40 ± 14.9^b^	295.60 ± 26.6^c^	165.80 ± 25.5^b^	619.20 ± 48.5^a^	354.20 ± 30.9^c^	167.80 ± 18.2^b^	0.0002

The analysis was performed with the Kruskal–Wallis tests and data are shown as Mean ± SEM.

^a,b,c,d^Different superscript letters indicate significant statistical difference (*P* ≤ 0.05).

The stimulation proliferation (S.I.) index was determined by CFSE dilution and the geometric mean values of the concanavalin A-stimulated duplicates were calculated and divided by the geometric mean values of the PBMC-nonstimulated control duplicates.

In relation to the CD3e^+^CD8α^+^ cytotoxic T cell population in the thymus (*P* < 0.009), spleen (*P* < 0.05), and mLN (*P* < 0.009) at the 24-h mark, the piglets that were allowed to suckle their mother’s colostrum (GG and SS groups) showed a significantly higher amount of cytotoxic T cells than the piglet groups that either suckled other mother’s colostrum (GS and SG) or were fed with milk replacer (GMR and SMR) (SG ˃ SS ˃ SMR and GS ˃ GG ˃ GMR groups) (Table 1). Similarly, CD3e^+^CD8α^+^CD27^+^ memory T cells in the spleen, thymus, and mLN were higher in the GG and SS groups than in the GS, SG, GMR, and SMR groups, though this was only significant in the thymus (*P* < 0.009).

We also evaluated the CD19^+^CD79a^+^ B cell (SWC7^+^CD79a^+^) populations of lymphatic tissues. When the piglets were fed with sow colostrum, a higher concentration of B cells was observed in these piglets (SS and GS groups), in splenocytes (*P* < 0.05) and thymocytes (*P* < 0.01). However, B cells from the mesenteric lymph node were higher in piglets born from sows (SS, SG, and SMR) rather than gilts (GG, GS, and GMR) (*P* < 0.05). Furthermore, piglets that received sow colostrum had a higher amount of B cells than their siblings that were fed either gilt colostrum or milk replacer (SS ˃ SG ˃ SMR and GS ˃ GG ˃ GMR groups) (Table 1).

Data for age-related changes in the transcription factor expression of T and B cells are shown in Table 2. As expected, a considerable increase of cell population with age was observed. Similar to the observations at 24 h in different organs, Concanavalin A stimulation tended to show an increase in helper T cell (CD3e^+^CD4^+^), naive and/or central memory T cell (CD3e^+^CD4^+^CD27^+^) and B cell (CD19^+^CD79a^+^) population in piglets that suckled sow colostrum compared to sibling piglets that suckled gilt colostrum and fed milk replacer (SS ˃ SG ˃ SMR and GS ˃ GG ˃ GMR groups). However, the populations of CD3e^+^CD4^+^CD25^+^ and CD3e^+^CD8a^+^CD27^+^ T cells were higher in piglets that were kept with their own dam and allowed to suckle normally (SS ˃ SG ˃ SMR and GG ˃ GS ˃ GMR groups) (*P* < 0.05). CD3e^+^CD8a^+^ T cells isolated from the thymus and spleen showed an increase in piglet groups that suckled other dam’s colostrum (SG ˃ SS ˃ SMR and GS ˃ GG ˃ GMR groups). The number of monocytes/macrophages in the spleen, thymus, and mesenteric lymph node tended to be higher in piglets from the sow colostrum groups (SS, SG, and SMR) than in the gilt groups, but the difference was not significant.

**Table 2:** Stimulation proliferation index for lymphocytes from lymphoid organs during the weaning period (28 days of age).

Lympatic organ	Immune cells	Groups – 28 days							Pr > χ^2^
			GG	GS	GMR	SS	SG	SMR	
Spleen	B cells	SWC7^+^CD79a^+^	3579 ± 162^b^	4996 ± 448^c^	3804 ± NA^b^	8462 ± 674^a^	7817 ± 587 ^a^	8074 ± 692^a^	0.0028
	T cells	CD3e^+^CD4^+^	8412 ± 1230^a^	12,366 ± 1400^b^	3112 ± NA^c^	11,752 ± 579^ab^	10,579 ± 1031^ab^	3974 ± 495^c^	0.0156
		CD3e^+^CD4^+^CD25^+^	610.40 ± 71.7^ab^	351.80 ± 32.7^c^	300.00 ± NA^c^	627.00 ± 51.5^a^	462.20 ± 30.5^bd^	341.33 ± 17.3^c^	0.0036
		CD3e^+^CD4^+^CD27^+^	293.60 ± 45,9^b^	693.80 ± 39.4^c^	170,00 ± NA^d^	564.20 ± 37,2^a^	391.40 ± 58.5^bd^	194.67 ± 12.6^d^	0.0020
		CD3e^+^CD8α^+^	751.00 ± 260^b^	2793 ± 304^ac^	827,00 ± NA^b^	1976 ± 152^ad^	2753 ± 322^c^	1844 ± 97^d^	0.0023
		CD3e^+^CD8α^+^CD27^+^	141.40 ± 11,4^a^	106.60 ± 16,4^b^	29.00 ± NA^c^	223.40 ± 34.8^a^	155.60 ± 23.3^a^	150.67 ± 48.2^a^	0.1289
	Myeloid cells	Macrophage^+^	1687 ± 50^b^	1475 ± 63^c^	320,00 ± NA^d^	2146 ± 106^a^	1814 ± 44^b^	1317 ± 94^c^	0.0012
Thymus	B cells	SWC7^+^CD79a^+^	3532 ± 394^b^	4315 ± 528^b^	4163 ± NA^b^	8954 ± 858^a^	8034 ± 605^a^	5237 ± 606^b^	0.0026
	T cells	CD3e^+^CD4^+^	3255 ± 79^b^	4002 ± 144^c^	3061 ± NA^b^	10,266 ± 814^a^	3821 ± 92^c^	3172 ± 265^b^	0.0015
		CD3e^+^CD4^+^CD25^+^	426.20 ± 25.2^a^	366.00 ± 13.9^b^	314.00 ± NA^b^	648.40 ± 113^a^	388.00 ± 65.7^b^	269.33 ± 39.7^b^	0.0493
		CD3e^+^CD4^+^CD27^+^	100.20 ± 3.72^b^	278,60 ± 61.1^bc^	73,00 ± NA^b^	647.20 ± 23.1^a^	241.40 ± 14.6^c^	73.67 ± 4.91^d^	0.0015
		CD3e^+^CD8α^+^	2702 ± 201^b^	3722 ± 127^ac^	2028 ± NA^b^	3329 ± 175^a^	3836 ± 113^c^	3164 ± 301^ab^	0.0114
		CD3e^+^CD8α^+^CD27^+^	779.00 ± 33.7^b^	517.00 ± 114^c^	430.00 ± NA^c^	5372 ± 175^a^	3331 ± 877^d^	1136 ± 75^d^	0.0012
	Myeloid cells	Macrophage^+^	355.60 ± 122^b^	498.00 ± 90.3^b^	259.00 ± NA^b^	650.80 ± 230^b^	745.40 ± 221^b^	99.33 ± 33.9^a^	0.0684
mLN	B cells	SWC7^+^CD79a^+^	4338 ± 707^ab^	4648 ± 221^a^	3840 ± NA	4723 ± 103^a^	4642 ± 223^a^	3019 ± 412^b^	0.0685
	T cells	CD3e^+^CD4^+^	2904 ± 143^b^	3106 ± 158^b^	6370 ± NA	4752 ± 400^a^	3498 ± 275^b^	2573 ± 702^b^	0.0245
		CD3e^+^CD4^+^CD25^+^	486.80 ± 41.9^b^	388.00 ± 35.7^c^	113.00 ± NA	1145 ± 97^a^	731.00 ± 27.5^d^	322.33 ± 74.0^c^	0.0015
		CD3e^+^CD4^+^CD27^+^	296.80 ± 21.1^b^	315.20 ± 5.44^b^	387.00 ± NA	595.00 ± 15.2^a^	438.00 ± 34.8^c^	361.00 ± 23.8^bc^	0.0019
		CD3e^+^CD8α^+^	2131 ± 129^b^	2354 ± 96^b^	987.00 ± NA	4163 ± 89^a^	2203 ± 160^b^	1289 ± 291^c^	0.0032
		CD3e^+^CD8α^+^CD27^+^	661.40 ± 26.8^a^	346.00 ± 35.2^c^	153.00 ± NA	815.00 ± 75.1^a^	560.20 ± 22.0^d^	408.67 ± 146^ cd^	0.0059
	Myeloid cells	Macrophage^+^	278.00 ± 21.0^b^	369.00 ± 43.2^bc^	341.00 ± NA	1797 ± 152^a^	836.80 ± 82.6^d^	506.00 ± 69.3^c^	0.0011

The analysis was performed with the Kruskal–Wallis tests and data are shown as Mean ± SEM.

^a,b,c,d^Different superscript letters indicate significant statistical difference (*P* ≤ 0.05).

The stimulation proliferation (S.I.) index was determined by CFSE dilution and the geometric mean values of the concanavalin A-stimulated duplicates were calculated and divided by the geometric mean values of the PBMC-nonstimulated control duplicate.

## Discussion

The immunoglobulins present in the colostrum are an important component for the initial immunological defense of the newborn, given that in pigs this is the only source of maternal antibodies for the litter. The concentration of IgG in the colostrum of females in the study was a mean 83.51 mg/mL, demonstrating that this was a good quality colostrum, considering a good quality level between 50 and 80 mg/mL of IgG during the first hours after labor^29^. IgG is the most common type of immunoglobulin present in the colostrum and almost 100% of it derives from the sow’s serum^30^, which transfers from the blood to the mammary gland by means of the FcRn receptor during colostrum genesis^31,32^.

The secretion of IgA and IgM in the colostrum is mediated by the polymeric Ig receptor (pIgR) in the mammary gland^33^. IgA is the second most present type of immunoglobulin in the colostrum and is predominant in the milk, with 40% originating in the blood and most produced in the mammary gland during lactation. However, considering that a high proportion of IgM and all IgG are derived from blood, consequently, almost 90% of immunoglobulins are of blood origin^30^. Although the concentration of IgM in the colostrum observed in the present study did not present differences between gilts and sows, the IgG content was higher in sows, which is consistent with previous results^30,34–36^. However, this was not reflected in the concentration of IgA in the total colostrum of the current study, with no differences between primiparous and multiparous sows, contrasting with several recent studies^35,36^ but with similar results to previous studies of our group^26^. These differences may be due to responses to nutritional changes^37^, as well as the genetic advances and moments when the colostrum samples are collected^38^.

The concentration of IgG in the colostrum is known to be highly variable among sows; therefore, genotype, parity, age, vaccination stage and endocrine status of the sow, nutrition, and herd management influences the yield and composition of the colostrum^37,39–41^. The concentration of IgG in maternal colostrum significantly affects acquired immunity and, therefore, knowledge about IgG content in the colostrum using precise measurements is crucial for adequate management when seeking to decrease pre-weaning mortality among piglets. The concentration of IgG in swine colostrum can vary between 48.0 and 95.6 mg/mL^35,42^. Due to the influence of parity in the concentration of colostrum immunoglobulins^43^, it is expected that piglets that are fed colostrum from sows receive a higher concentration of immunoglobulins than those that are nursed by gilts. Thus, we observed in the present study a difference in IgG and IgM serum concentration among sibling piglets. This variability was a consequence of the type of colostrum that these siblings received; we observed a higher concentration in the SS group than in the SG group, and the GS group had a higher immunoglobulin concentration than the GG group. In the present study, piglets that received colostrum from multiparous sows presented higher serum concentrations of IgG, IgA, and IgM than piglets that received colostrum from gilts. Moreover, the sibling piglets that received milk replacers presented a “negative” concentration of IgG and IgM during their first 24 h of life and showed a lower concentration than their siblings that received colostrum (GG, GS, SS, and SG)^23^.

The intestinal mucosa is very sensitive to dietary stimuli, so piglets fed only with milk replacers since birth have an early cessation of macromolecule endocytosis^28^. This clarifies the reason why piglets in these groups (GMR and SMR) of the present study did not present any IgG titer in their serum, even once they were returned to their mothers after 24 h of life, thus suggesting that intestinal absorption and closure had already happened. For the same reason, we observed that piglets that suckled gilt colostrum had lower levels of IgA at birth because the IgA concentration in gilt colostrum had a lower absolute value than that of sows, even without statistical difference between sows and gilts^34^. Piglets deprived of colostrum ingestion in the first 24 h of life and/or fed only with milk replacer since birth did not present immunoglobulin titers in their serum after completing these first 24 h of life (groups GMR and SMR), demonstrating that the piglets have agammaglobulinemia at birth and that the only way to acquire immunoglobulin is through the colostrum, thus corroborating several other studies^3,4,28,44^. However, the differences in IgA between piglets that suckled gilt colostrum and the IgG and IgM in piglets fed with milk replacer (GMR and SMR groups) at 28 days of life were reversed, probably due to higher de-novo synthetization of Ig in these piglets^34^. Fetal and newborn piglets use primarily four VH genes and two DH segments to generate their preimmune natural antibody repertoire^45^. The pre-immune repertoire is often limited and may develop in the absence of environmental antigenic stimulation. Moreover, newborn mammals have B lymphocytes and small amounts of de novo synthesized antibodies that are encoded by early rearrangements and developmental events^46^. Thus, colonization of the gastrointestinal tract in mammals affects the development of the mucosal immune system^47^ and also appears to stimulate diversification of the preimmune repertoire with increases in the transcription of IgA and serum IgA levels^48^. Therefore, we predicted that the agent responsible for IgA induction in piglets that suckled gilt colostrum was orally introduced, synthesized by the piglets themselves^7^. It is important to note that maternal IgA from the milk did not contribute to the level of neonatal serum IgA^49^.

During the study, eight animals died. They all died during the period between 5 and 20 days of life, considering that animals were assessed for a period of 28 days. These eight animals belonged to the group that received the milk replacer (five animals from GMR and three from SMR). The piglets had no clinical signs of infection, fever, or diarrhea during the nursing period. Diarrhea was observed during and shortly after the use of the milk replacer, i.e., was observed in the first 2 days of life. Due to the high mortality observed in these animals during the pre-weaning period, we chose to not use more animals since the diet they received during the first 24 h of life left them immunocompromised, according to the immunological assessments carried out among the few surviving animals of the group. In fact, IgG is the clinically most important type of immunoglobulin during these first weeks of life, and the concentration of IgG in piglet plasma after birth is positively correlated with survival. Moreover, the dead piglets had a lower IgG serum concentration than the surviving piglets, indicating that colostrum had not been absorbed, even once they were returned to their mothers and nursed with maternal milk after 24 h^40,50^. It is important to mention that piglets become immunocompetent after 4 weeks of age^1,51^. Therefore, reduced growth among the litter and mortality of healthy offspring can be considered a consequence of immunologically poor colostrum production^34^, given that IgG content in colostrum varies greatly among sows^30,39^.

The serum concentration of immunoglobulins in piglets also varies greatly between herds and between sows^52^. The present results corroborate that there is a strong association between the IgG concentration found in colostrum and the serum concentration of IgG in the piglet. This demonstrates that an increase in the IgG level of colostrum improves IgG levels in piglets and could potentially increase their survival rates^35^. Paying extra attention to small piglets in a non-homogeneous litter may be necessary to guarantee sufficient IgG absorption^53^. IgG capture can be measured indirectly by registering IgG levels from piglet serum after birth ^54^. There is also the possibility that low immunoglobulin serum concentrations among piglets may result from absorption failures, which is related to the intestinal maturation of the piglet. However, the amount of colostrum consumed by piglets and the amount of immunoglobulin presented to the intestine for absorption is unknown^55,56^.

Finally, absorption efficiency decreases when the ingestion of the first colostrum is delayed, indicating the importance of ingesting colostrum immediately after birth. Even more important is the transmigration of pathogenic bacteria that can be avoided with colostrum by “closing the intestine”^2,12,57^ in the intestinal lumen. In cattle, the absorption efficiency of IgM decreases as ingestion increases, such that the ingestion of a greater amount of IgM does not increase the absolute amount that is absorbed^58^. The results regarding levels of IgM in the milk replacer group at 28 days suggest that the evolution in concentration of this immunoglobulin is related to an immunity maturation in the piglets, considering that these animals did not ingest colostrum.

The immunology of the intestinal mucosa is highly complex, given that the surface of the mucosa is not only a predominant factor in the structural and immunological barrier against microorganisms, but also has an important role in the absorption of water and nutrients during the digestive process^59^. In fact, the epithelium of the intestinal villi has an important role in the absorption of colostrum IgG, and likely IgA and IgM for neonatal circulation, and a specialized role for the epithelium of the intestinal crypt in the adsorption of colostrum IgA and IgM^60^. It is important to note that dimeric IgA and pig colostrum IgM offer a “first antiseptic layer” for the intestine, avoiding the transmigration of pathogenic bacteria^2,60,61^. Moreover, we observed in the present results that piglets deprived from the ingestion of colostrum in the first 24 h of their lives and fed only with milk substitutes during the colostrum phase presented high serum concentrations of IgA at 24 h of life, which were significantly higher than the concentrations observed for piglets in the groups that received colostrum. Levast, et al.^61^ suggest that ultra-early weaning directly influences the synthesis of IgA, activates, directly or indirectly, the Th17 pathways, and may be related to alterations in the functions of the mucosa barrier. On the other hand, a delay in weaning allows that endogenous factors of the milk to stimulate the beginning of IgA synthesis, protecting the piglet. Thus, we suggest that the milk substitute stimulated an early synthesis of IgA by the piglet itself. It is likely that a component of the milk substitute acted as an antigen and was transferred to duodenal M cells for the intraepithelial lymphocytes, stimulating the plasma cells of the mucosa to secrete IgA^59^. This increase was transient and was only observed while the animals received the milk replacement. In the lactogenesis phase, no difference was observed among the piglets.

As demonstrated in our study, corroborating previous efforts, no artificial nursing system using formula and milk replacers can reproduce the functions and characteristics in a pig’s organism as maternal colostrum^62^. In this context, the study also assessed the influence of different diets in the first 24 h of life and cell immune development in piglets.

In this study, colostrum and plasma cytokines were measured in dams and their offspring newborns throughout the first day of life, using a quantitative multiplex protein assay. It is assumed that cytokines are absorbed into the offspring’s circulation before gut closure takes place and mammary secretion changes from colostrum to mature milk. Our understanding of early life ontogeny of piglet plasma cytokine and chemokine concentrations is limited. In humans, these concentrations can be affected by maturation as well as infectious and/or inflammatory states^63^. Colostrum and plasma concentrations of Th1, Th2 or Th17 cytokines were higher in sows than in gilts, and piglets that suckled sow colostrum also showed higher concentrations of these cytokines in their plasma than piglets that suckled gilt colostrum and piglets fed milk replacer. The production of GM-CSF, IFNγ, IL-1α, IL-1β, IL-2, IL-4, IL-6, IL-10, IL-12, IL-18, and TNFα was higher in all piglet groups that suckled sow colostrum. But even the groups of piglets that suckled gilt colostrum produced less than half of the amount of GM-CSF, IFNγ, IL-1α, IL-1β, IL-2, IL-4, IL-6, IL-10, IL-12, IL-18, and TNFα as compared to the SS and GS groups (sow colostrum). This lower cytokine concentration was the same for piglet groups fed milk replacer. Piglets showed higher colostrum-related innate cytokine responses (IL-1β, IL-6, TNF-α, and IL-10) in the case of multiparous dams. This innate maturation corresponds to a parallel increase in adaptive Th1 (IFN-γ) responses to mitogens.

Th cells are a type of T-lymphocyte and their proliferation is able to turn them into effector T-cells, which subsequently differentiate into two distinctive subtypes, Th1 and Th2-lymphocyte cells^64^. Moreover, Th cells either enhance or potentiate the activity of other immune cells by releasing T cell cytokines. Th1-supporting IL-2, IFN-γ, and TNF-α cytokine production are involved in cellular immunity, while Th2 lymphocytes characterized by the production of IL-4, IL-5, IL-10, and IL-13 are mainly involved in humoral immunity. Th17 lymphocytes are known to produce IL-6^65^.

We then examined the relationship between early innate immune responses and the source of feed in suckling piglets. A greater propensity for innate inflammatory and anti-inflammatory responses in the perinatal period was significantly correlated with piglets that suckled sow colostrum, represented by the SS and GS groups, along with propensity for specific Th1 and Th2 responses in the early postnatal period. Specifically, there were consistent positive correlations between the level of inflammatory cytokine responses at 24 h of life and the development of IL-6, IL-12, IL-18, and IL-β, but also for TNF-α. This was also particularly true for anti-inflammatory cytokine IL-10 production. In contrast, piglets fed milk replacer were associated with significantly increased concentrations of IL-1ra and IL-8. The IL-1 receptor antagonist (IL-1ra) is an anti-inflammatory agent that reduces inflammation by blocking the binding of the agonist receptor ligands^66^. Some properties of the milk replacer composition may be antigenic and supplementary analyses may be useful to detect the degree of immune reactivity against antigens of these products. We thus observed that sow parity is correlated with colostrum and plasma cytokine concentrations. Colostrum and plasma cytokine concentrations from the dam improve the immunological quality of the colostrum with an immunomodulatory effect and is a strong driver of porcine neonatal ontogeny.

After the piglet is born, the immune system begins to transition towards a microbe-rich extra-uterine environment. Nevertheless, the development of active cellular immunity in the piglet is immature at birth^67^. The ability of B and T cells from lymphatic organs and blood mononuclear cells to respond to mitogens was less developed in the neonate piglet and the number of antigen-presenting cells was lower, which is consistent with other studies^9^. The capacity for active proliferation and clonal expansion of T and B cells, which is required for the generation of adaptive immune responses, was observed at different levels when considering the piglet groups that suckled on sow colostrum and on gilt colostrum, and fed milk replacer; activated B and T cells were higher in piglets breastfed with sow colostrum in the neonatal period (24 h) and then with sow mature milk in the weaning period (28 days)^7^.

We do not yet know whether tolerance can be generated in offspring by additional antigens from components of either another dam’s colostrum or milk replacer. We did not observe an increase in regulatory T cells with cytokine production in piglets that either suckled another dam’s colostrum or were given milk replacer, which induce the production of regulatory cells that may survive as long-lived memory T cells. Our findings showed higher CD4^+^CD25^+^ Treg and CD3e^+^CD8a^+^CD27^+^ T memory/cytotoxic cells in piglets that were kept with their own dam and allowed to suckle as normal, by 28 days of age. The data suggest an accelerated development of these subsets of lymphocytes in these piglets. The functional significance of these changes is unclear because the cell surface markers used in this study do not provide information regarding the level of activity of these cells. Indeed, CD4^+^CD25^+^ Treg cells play a vital role in peripheral tolerance and are lower in allergic animals^68^. Several studies have shown that there is an expansion of CD4^+^CD25^+^ Treg cells in Peyer’s patches, LnM, and peripheral lymphoid tissues of mice treated orally with antigens^69^. The regulatory activity of CD4^+^CD25^+^ Treg cells is linked to the production of Th2 cytokines, such as IL-4 and IL-10, and associated with the suppression of the production of Th1 cytokines, such as IL-2 and INF-γ. These results indicate that nutrition influences the functional capacity and maturation of the T cell population. Thus, the components of the early-life environment and nutrition affects both local development of regulatory components of the mucosal immune system and immune responses to food proteins from milk replacer. Clearly, development of immunocompetence is an absolute requirement for optimum growth and performance. However, in the context of exposure to a wide range of antigens associated with pathogens, a definition of immunocompetence must consider the ability to mount appropriate responses to antigens. This will include the ability to generate tolerance to food and commensal bacterial antigens as well as active immune responses to pathogens.

The pigs fed with milk replacer (GMR and SMR groups) showed some lymphocytes that were unable to respond to mitogens between 24 h and 28 days, suggesting that immune cells are mainly immature, whereas the piglets that suckled sow/gilt colostrum showed greater amounts of mitogen-stimulated lymphocytes^7^. We observed that the IgG concentrations in piglet plasma shortly after birth are positively correlated with survival^70^ and, in addition, dead piglets had lower serum IgG concentrations than comparable surviving piglets^2,71,72^. Our study supports findings regarding immune deficiency for immune cells and cytokine production in neonates, as demonstrated in piglets fed with milk replacer. In addition, our study is also the first to document the influence of sow colostrum on the increase of activated B and T cells, as well as pro-inflammatory/anti-inflammatory cytokine secretion capacity that evolves parallel to Th1 and Th2 maturation over time. Naive T cells do not secrete Th1, Th2, and Th17 cytokines, thus, changes in cytokine concentrations secreted by either Th1 or Th2 cells are associated with altered proportions of lymphocyte subpopulations and antigen-driven T cell differentiation.

The trajectories between the first week of life and the remainder of the piglet’s vulnerable neonatal period, defined as the first 28 days of life, showed that immune ontogeny does not remain consistent. However, our results demonstrated that animals that were fed with milk replacer had a higher mortality rate than piglets that were fed colostrum. Colostrum plays a major role in the survival of newborn piglets, and studies about the relationship between colostrum and the ontogeny of immune reactions are important to understand the immunological process and the possible factors that could compromise this process. Indeed, our findings also demonstrated that sow parity influences the immunomodulatory effect of colostrum and impacted amounts of mitogen-stimulated lymphocytes. Whether this early impact on the piglet’s life will affect performance is a key question in the field of immune ontogeny and will require further studies. The findings demonstrated that the relative and absolute sizes of lymphocyte populations vary with age during the lactation period because of the maturation of the piglet’s immune system during this period. We expect our findings can help define the baseline trajectories of plasma cytokines in neonate piglets and will help to correlate plasma, in future studies, and cytokines with immune response challenges, such as immunization. Lastly, future studies on the influence of sow parity in colostrum and its impact on either mucosal immunocompetence or the gut microbiome of piglets will provide a tool for the implications under practical management conditions in the first hours of a piglet’s life.

## Materials and methods

### Ethics statement

The protocols and the use of animals for this research were approved by the Ethics Committee on Animal Use (CEUA) from the Embrapa Swine and Poultry National Research Center under protocol # 001/2016. This study was conducted in compliance with the ARRIVE guidelines. All methods were carried out in accordance with relevant guidelines and regulations.

### Animals

This study was conducted on nine crossbred Landrace 3Large White (LR3LW) dams. All sows were housed individually on a slatted floor during gestation. Seven days before the expected farrowing, dams were transferred from the gestation to the farrowing room. Sows had free access to water and were fed twice a day on a traditional gestation diet. The gestation diet was provided until the second day of lactation.

A total of 30 candidate healthy dams (20 sows and 10 gilts) with similar expected delivery dates were selected and prostaglandin F2 alpha was administered at 6:00 AM on day 113 of gestation to ensure synchronous delivery. Candidate sows/gilts were excluded if the difference in delivery time was more than 1 h. In total, five synchronously delivering adult pregnant multiparous sows (parity = 4∼6) and four primiparous gilts were selected for this study. All sows were examined (feed intake, rectal temperature, vulval discharge, and milk production) to exclude the possibility of puerperal disorders, mainly mastitis-metritis-agalactia syndrome. The day of parturition (just after the birth of the first piglet) was considered day 0 (D0) of the experiment. Some piglets from these nine dams remained with their dam, while others were relocated. Thus, six groups were formed:

- GG group: piglets were kept with their own gilts and allowed to suckle as normal (gilt colostrum, n = 10);
- GS group: piglets were changed from gilts to sows and allowed to suckle as normal (sow colostrum, n = 10);
- GMR group: piglets from gilts were isolated from the dams, kept in containers under controlled temperature and were deprived of colostrum (negative group). Piglets were bottle-fed commercial formula milk replacer for pigs (Vetmilk S, Agrifirm-Brazil) every 1 h during the first 24 h of life (n = 6).
- SS group: piglets were kept with their own sows and allowed to suckle as normal (sow colostrum, n = 10);
- SG group: piglets were changed from sows to gilts and allowed to suckle as normal (gilt colostrum, n = 10);
- SMR group: piglets from sows were isolated, colostrum-deprived, and placed in the same condition as the GMR group (n = 8).

**Figure 7:**
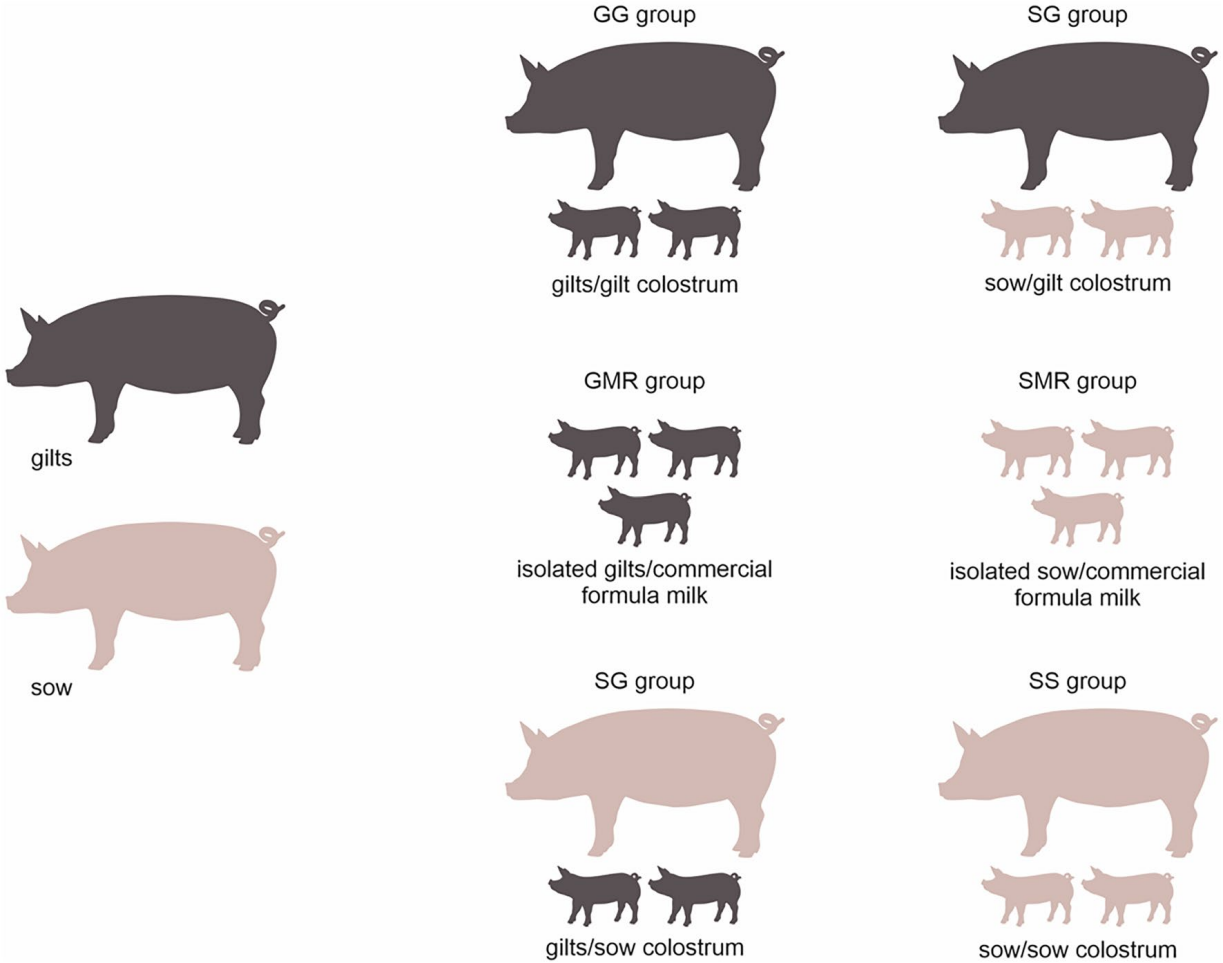
Experimental design: At equal proportions, part of the piglets born to a sow suckled on a sow of the same parity, while another part suckled on a gilt, and another part was fed milk replacer for 24 h. The same was done for the piglets that were born from the gilts. All cross-fostering required was done immediately after birth and before the first colostrum intake.

The piglets remained in the groups during the first 24 h of life and were later returned to their respective mothers (Fig. 7). Suckling and milk replacer were provided ad libitum. Blood (serum clot activator tubes and heparinized tubes) and colostrum samples were collected from the sows on D1 postpartum. Piglet serum and whole blood (heparinized tube) samples were collected on D1 and D28 after birth. Serum was collected and stored at -80 °C until further analysis. Peripheral blood mononuclear cells (PBMCs) were isolated from the heparinized blood samples a few hours after collection. Colostrum was manually collected from all functional teats, after the birth of the first piglet (D0) and before suckling. To minimize the contamination of colostrum and milk, the sows’ teats were previously scrubbed with iodine alcohol, handled wearing disposable latex gloves and samples were stored in sterile 50 mL conical tubes (TPP; Switzerland) and kept refrigerated^73^. Colostrum and samples were centrifuged for 20 min (1300xg at room temperature), and the upper fat layer was discarded. After the initial treatment, serum and colostrum samples were stored at -80 °C for further analysis. On D1 and D28, the piglets from all groups were euthanized at each timepoint. During the necropsy, the spleen, thymus, and mesenteric lymph node were collected in a RPMI 1640 medium (Gibco, USA) supplemented with 100 U of penicillin G (Sigma-Aldrich, USA), and 0.1 mg/mL streptomycin and 0.25 µg/mL of fungizone (pH 7.4, Thermo Fisher Scientific, USA).

### Immunoglobulin and cytokine quantification in colostrum and serum samples

IgA, IgG, and IgM titers in colostrum and serum samples were determined using ELISA kits for porcine IgA, IgG, and IgM quantification (Bethyl Laboratories, Inc., USA), which were quantified according to Forner et al.^24^. The concentrations of 13 cytokines were measured in undiluted colostrum and serum samples by Porcine Cytokine/Chemokine Magnetic Bead Assay (MILLIPLEX®MAP, Cat#PCYTMG-23 K-13PX, Merck Millipore, USA), following the manufacturer’s recommendations. The quantified cytokines/chemokines were the granulocyte macrophage colony-stimulating factor (GM-CSF), interferon-gamma (IFNγ), IL-1α, IL-1β, IL-1ra, IL-2, IL-4, IL-6, IL-8, IL-10, IL-12, IL-18, and the tumor necrosis factor-α (TNFα). The plates were run on a MAGPIX® analyzer (Luminexcorp, Austin, TX, USA). The software xPONENT version 4.2 (Luminexcorp) and MILLIPLEX® Analyst Version 5.1 (Merck Millipore, USA) were used for data acquisition and analysis, respectively. The median fluorescence intensity, analyzed using a 5-parameter logistic curve fit, was used to calculate cytokine concentrations, which were expressed in ng/mL.

### Isolation of peripheral blood mononuclear cells (PBMCs)

Heparinized blood samples were diluted 1:3 (v/v) in PBS and the PBMCs were isolated through density gradient centrifugation (Ficoll-Paque, GE Healthcare; Sweden), following the manufacturer’s recommendations. The PBMCs were cryopreserved in 95% fetal bovine serum (FBS; Gibco; Brazil) + 5% Dimethyl sulfoxide (DMSO; Sigma-Aldrich; United Kingdom).

### Isolation of white-blood cells from the spleen, thymus, and mesenteric lymph node

The piglets were euthanized and the spleen, thymus, and mesenteric lymph nodes from each piglet at predetermined time points (D1 and D28) were isolated under aseptic conditions. The isolated tissues were individually disrupted with the plunger of a 1 mL syringe against a 100 µm cell strainer to get single-cell suspension and collected in Hanks’ balanced salt solution (HBSS, Gibco, Brazil) + 2% FBS. Then, the cells were filtered to avoid tissue debris by passing cell suspension through a 70-μm Nylon Cell strainer, and were centrifuged in single-cell suspensions at 300xg for 10 min at 4 °C. After that, the cells were resuspended with Pharm Lyse™ buffer (BD Biosciences, USA) to lyse red blood cells at room temperature. The lysis reaction was stopped by adding 10 volumes of RPMI 1640 medium + 2% FBS and centrifuged at 300xg for 10 min at room temperature. The cells were washed twice, then the supernatant was discarded, the cells were resuspended in complete RPMI 1640 medium, and the cell number was counted with 0.4% Trypan blue to determine the viable cell concentration. The cells were resuspended and cryopreserved in 95% FBS + 5% DMSO at a final concentration of 2 × 10^6^ cells/mL.

### Cell proliferation assay

The use of carboxyfluorescein diacetate succinimidyl ester (CFSE) in combination with monoclonal antibodies (mAbs) enabled the concomitant determination of cell proliferation and activation status of cell subpopulations with a protocol adapted from Chaoul et al.^74^. The viable cells from the spleen, thymus, mesenteric lymph node, and PBMCs were thawed and suspended to the concentration of 5 × 10^6^ cells/mL in Dulbecco’s phosphate-buffered saline (DPBS, Sigma-Aldrich, USA) and labeled with CFSE (2.5 μM; CellTrace CFSE Cell Proliferation kit, Invitrogen, USA) for 15 min at 37 °C in the dark. Then the labeling process was stopped by the addition of ice-cold RPMI 1640 supplemented with 10% FBS, followed by incubation for 5 min in an ice bath, in the dark. Finally, the cells were washed twice with RPMI-FBS and further suspended in complete RPMI 1640 medium, this medium was supplemented with 10% FBS (Gibco, Brazil), 1 mM GlutaMAX (Gibco, Brazil), 25 mM HEPES (Sigma-Aldrich, USA), 1 mM sodium pyruvate (Sigma-Aldrich, USA), 50 M 2-mercaptoethanol (Gibco, USA) and 100 U/mL penicillin–streptomycin (Sigma-Aldrich, USA).

The cells were plated in 24-well plates (5 × 10^6^ cells/well) and cultured for 96 h with complete RPMI 1640 medium at 37 °C under 5% CO2, and in vitro mitogens stimulation occurred with 5 µg/mL of Concanavalin A from *Canavalia ensiformis* (Sigma-Aldrich, USA)^75^.

After 96 h of culture, the cells from lymphatic organs (spleen, thymus, and mesenteric lymph node) were visualized using microscopy (EVOS M7000, Thermo Fisher Scientific), then were quantified, suspended in a flow cytometry buffer (1 × 10^6^), transferred to flow cytometry tubes, and labeled for 30 min at room temperature with a cocktail of specific mAbs. For the flow cytometry, we used antibodies raised against porcine leukocyte antigens and isotype controls (BioRad Serotec, Oxford, UK), stabilizing fixative, and compensation beads from BD (North Ryde, Australia). Our flow cytometry panel was carried out following a 4-color panel to assess lymphocyte and monocyte-macrophage cell populations. The following fluorochrome-labeled mAbs were used: panel A): 7-AAD (BD Biosciences, USA), RPE-macrophages (clone BA4D5); panel B): RPE-CD4alpha (clone MIL17), PE-Cy7-CD8alpha (clone MIL12), APC-CD3 (clone PPT3); panel C): RPE-CD4alpha (clone MIL17), PE-Cy7-CD25 (clone K2313B2), APC-CD3 (clone PPT3); panel D): RPE-CD4alpha (clone MIL17), PE-Cy7-CD3 (clone PPT3), APC-SWC2 (or CD27, clone B30C7); panel E): RPE-CD8alpha (clone MIL12), PE-Cy7-CD3 (clone PPT3), APC-SWC2 (or CD27, clone B30C7); panel F): PE-Cy7-SWC7 (or CD19, clone CC55), RPE-CD79a (clone MB-1). The following were used for PBMC: panel A): 7-AAD (BD Biosciences); panel B): RPE-CD79a (clone MB-1), PE-Cy7-SWC7 (or CD19, clone CC55); panel C): RPE-CD4alpha (clone MIL17), PE-Cy7-CD8alpha (clone MIL12), APC-CD3 (clone PPT3).

For intracellular staining, the cells were treated with the Cytofix/Cytoperm Fixation/Permeabilization kit (BD Biosciences, USA), following the manufacturer’s instructions, and stained with CD79a, macrophages, and CD3 mAbs. The antibodies used in the staining were previously titrated for their optimum concentrations^24^. A total of 50,000 events per tube was acquired on the flow cytometer (Accuri C6plus and FACSCanto, Becton–Dickinson, USA) and analyzed with the aid of the software FlowJo (Becton–Dickinson, USA). The lymphocyte and macrophage gates were set on light-scatter properties (forward scatter vs. side scatter). Proliferation by CFSE (reflected by successive decrease of fluorescence intensities by dye distribution to daughter cells) was measured by flow cytometry. The results were expressed as counts of stained cells.

### Statistical analysis

Comparisons among groups were carried out using the Kruskal–Wallis test. Statistical analyses were carried out using the NPAR1WAY procedure of SAS (2012). Data are expressed as the mean ± standard error. Significance was declared at *P* ≤ 0.05.

## Supplementary Information

Supplementary Information.

## Data Availability

The data that support this study are available from the corresponding author upon reasonable request. Source data are provided with this paper.
