# Supplementary material for: On the influence of the source of porcine colostrum in the development of early immune ontogeny in piglets
Source: Sci Rep. 2022 Sep 17;12:15630. doi: 10.1038/s41598-022-20082-1 (PMC9482628; doi:10.1038/s41598-022-20082-1)
Supplement: Supplementary file 1 — Supplementary Information. [file 41598_2022_20082_MOESM1_ESM.docx]

**Table S1.** Body weight gains in piglets over the first day of life

|  | **Groups** | | | | | | | **Pr>χ^2^** | |
| --- | --- | --- | --- | --- | --- | --- | --- | --- | --- |
|  | **GG** | **GS** | **GMR** | **SS** | **SG** | **SMR** |  | |  |
| Birth weight, kg | 1.292±0.048 | 1.293±0.063 | 1.287±0.093 | 1.522±0.091 | 1.384±0.083 | 1.380±0.097 | 0.3820 | |  |
| 24 h weight, kg | 1.401±0.053 | 1.311±0.059 | 1.428±0.142 | 1.624±0.104 | 1.411±0.088 | 1.411±0.091 | 0.3596 | |  |
| Birth to 24 h weight gain, kg | 0.109±0.023 | 0.018±0.015 | 0.142±0.114 | 0.102±0.055 | 0.027±0.020 | 0.031±0.019 | 0.2838 | |  |

Error bars represent the standard error of the mean (± SEM). The analysis was performed with the Kruskal-Wallis tests.

**Table S2.** Cytokine concentrations (ng/ml) in colostral supernatant from gilt and sow.

| **Cytokines** | **Groups** | | **Pr>χ^2^** |
| --- | --- | --- | --- |
|  | **Gilt** | **Sow** |  |
| **GM-CSF** | 0.006±0.005 | 0.146±0.071 | 0.0399 |
| **IFN-y** | 7.178±5.993 | 47.05±21.3 | 0.0207 |
| **IL-1α** | 0.079±0.029 | 0.507±0.192 | 0.0195 |
| **IL1β** | 0.102±0.052 | 1.154±0.588 | 0.0093 |
| **IL-1RA** | 0.588±0.229 | 4.190±1.929 | 0.0226 |
| **IL-2** | 0.522±0.210 | 8.113±6.058 | 0.0272 |
| **IL-4** | 4.428±2.722 | 68.47±22.2 | 0.0225 |
| **IL-6** | 0.102±0.059 | 4.990±4.161 | 0.0147 |
| **IL-8** | 19.83±6.51 | 8.422±3.525 | 0.1454 |
| **IL-10** | 1.498±0.763 | 14.29±4.87 | 0.0076 |
| **IL-12** | 0.318±0.111 | 2.005±0.825 | 0.0173 |
| **IL-18** | 2.297±1.005 | 19.39±8.67 | 0.0272 |
| **TNF-α** | 0.077±0.027 | 0.589±0.305 | 0.0574 |

Data are shown as Mean ± SEM

**Table S3.** Concentration of plasma cytokines and chemokines (ng/mL) from gilt and sow.

| **Cytokines** | **Groups** | | **Pr>χ^2^** |
| --- | --- | --- | --- |
|  | **Gilt** | **Sow** |  |
| **GM-CSF** | 0.118±0.048 | 4.233±3.797 | 0.0037 |
| **IFN-y** | 4.011±2.496 | 243.93± 108 | 0.0076 |
| **IL-1α** | 0.181±0.088 | 0.708±0.210 | 0.0543 |
| **IL1β** | 0.424±0.248 | 2.020±0.654 | 0.0911 |
| **IL-1RA** | 1.113±0.565 | 5.577±1.769 | 0.0412 |
| **IL-2** | 1.056±0.630 | 9.900±4.405 | 0.0247 |
| **IL-4** | 26.69±25.2 | 120.97±36.1 | 0.0226 |
| **IL-6** | 0.378±0.333 | 3.920±1.559 | 0.0260 |
| **IL-8** | 0.050±0.013 | 0.252±0.160 | 0.5645 |
| **IL-10** | 6.716±5.703 | 28.73±.,96 | 0.0181 |
| **IL-12** | 0.843±0.342 | 3.170±0.900 | 0.0500 |
| **IL-18** | 5.261±3.313 | 37.56±13.5 | 0.0500 |
| **TNF-α** | 0.084±0.030 | 3.744±2.373 | 0.0593 |

Data are shown as Mean ± SEM.

**Table S4.** Levels of plasma cytokines (ng/mL) in piglet groups at 24 hours. Data are shown as Mean ± SEM.

| **Cytokine** | **Group** | | | | | | **Pr>χ^2^** |
| --- | --- | --- | --- | --- | --- | --- | --- |
|  | **GG** | **GS** | **GMR** | **SS** | **SG** | **SMR** |  |
| **GM-CSF** | 0.058±0.032 ^b^ | 0.746±0.278 ^a^ | 0.014±0.010 ^b^ | 0.918±0.452 ^a^ | 0.054±0.011 ^b^ | 0.012±0.012 ^b^ | 0.0004 |
| **IFN-y** | 33.44±7.50 ^b^ | 169.04±56.2 ^a^ | 4.084±2.033 ^c^ | 294.80±50.2 ^a^ | 22.01±10.6 ^bc^ | 13.37±6.99 ^bc^ | 0.0004 |
| **IL-1α** | 0.192±0.098 ^b^ | 0.604±0.108 ^a^ | 0.006±0.004 ^c^ | 0.744±0.146 ^a^ | 0.280±0.132 ^ab^ | 0.006±0.006 ^c^ | 0.0003 |
| **IL1β** | 0.382±0.222 ^b^ | 1.414±0.384 ^a^ | 0.012±0.002 ^b^ | 1.978±0.597 ^a^ | 0.636±0.358 ^b^ | 0.164±0.154 ^b^ | 0.0024 |
| **IL-1RA** | 13.79±3.51 | 10.48±2.90 | 34.29±22.5 | 5.912±1.436 | 22.89±8.74 | 30.48±26.9 | 0.3699 |
| **IL-2** | 1.462±0.586 ^b^ | 4.582±0.889 ^a^ | 0.100±0.044 ^c^ | 6.306±1.701 ^a^ | 1.886±0.357 ^b^ | 0.116±0.096 ^c^ | 0.0002 |
| **IL-4** | 24.54±14.7 ^b^ | 107.77±20.6 ^a^ | 0.086±0.048 ^c^ | 176.61±25.5 ^a^ | 52.62±11.5 ^b^ | 0.084±0.064 ^c^ | 0.0002 |
| **IL-6** | 0.434±0.256 ^b^ | 2.062±0.339 ^a^ | 0.042±0.027 ^b^ | 3.326±1.280 ^a^ | 0.636±0.376 ^b^ | 0.034±0.034 ^b^ | 0.0013 |
| **IL-8** | 0.242±0.051 ^c^ | 0.476±0.087 ^abc^ | 0.788±0.145 ^a^ | 0.512±0.087 ^ab^ | 0.228±0.060 ^bc^ | 0.764±0.275 ^abc^ | 0.0407 |
| **IL-10** | 6,896±3.721 ^c^ | 27.59±6.41 ^b^ | 0.224±0.088 ^d^ | 47.47±4.69 ^a^ | 13.72±2.76 ^c^ | 0.214±0.123 ^d^ | 0.0002 |
| **IL-12** | 0.996±0.320 ^b^ | 2.624±0.033 ^a^ | 0.362±0.027 ^b^ | 3.514±0.708 ^a^ | 1.134±0.486 ^b^ | 0.420±0.064 ^b^ | 0.0014 |
| **IL-18** | 7.086±3.473 ^bc^ | 24.02±2.80 ^a^ | 0.498±0.213 ^cd^ | 33.71±8.45 ^a^ | 9.458±4.280 ^b^ | 0.472±0.263 ^d^ | 0.0004 |
| **TNF-α** | 0.202±0.070 ^b^ | 2.348±0.833 ^a^ | 0.160±0.061^b^ | 3.044±1.190 ^a^ | 0.280±0.071 ^b^ | 0.130±0.076 ^b^ | 0.0008 |

The analysis was performed with the Kruskal-Wallis tests. ^a, b, c, d^ Different superscript letters indicate the significant statistical difference between (p≤0.05).

**Table S5.** Stimulation proliferation index for lymphocytes from peripheral blood among all groups at 24 hours and 28 days of age.

| **Immune Cells** | |  | **Piglet Groups** | | | | | | **Pr>χ^2^** |
| --- | --- | --- | --- | --- | --- | --- | --- | --- | --- |
|  |  |  | **GG** | **GS** | **GMR** | **SS** | **SG** | **SMR** |  |
|  | **24 hours** | | | | | | | | |
| **B cells** | | SWC7^+^CD79a^+^ | 292.6±40.6^cd^ | 562.8±33.1^b^ | 165.2±24.5^e^ | 724.1±37.2^a^ | 377.9±14.2^c^ | 199.7±6.8^de^ | <0.0001 |
| **T cells** | | CD3e^+^CD4^+^ | 193.13±19.2^de^ | 402.00±24.6^b^ | 219.33±8.74^d^ | 539.78±43.6^a^ | 269.00±11.6^c^ | 164.33±8.53^e^ | <0.0001 |
|  | | CD3e^+^CD8α^+^ | 74.63±4.25^d^ | 198.60±21.4^b^ | 78.50±9.50^d^ | 548.33±34.1^a^ | 137.50±4.33^c^ | 75.83±7.77^d^ | <0.0001 |
|  |  | | | | | | | | |
|  | **28 days** | | | | | | | | |
| **B cells** | | SWC7^+^CD79a^+^ | 1338±46^b^ | 1399±36^b^ | 224.00±NA | 2089±91^a^ | 1461±37^b^ | 1167±43^c^ | 0.0027 |
| **T cells** | | CD3e^+^CD4^+^ | 1569±32^c^ | 1723±74^bc^ | 1294±NA | 2750±77^a^ | 1886±63^b^ | 1627±53^c^ | 0.0020 |
|  | | CD3e^+^CD8α^+^ | 476.20±24.6^e^ | 982.40±31.5^c^ | 145.00±NA | 2404± 77^a^ | 1213±52^b^ | 616.00±30.5^d^ | 0.0005 |

The analysis was performed with the Kruskal-Wallis tests and data are shown as Mean ± SEM. ^a, b, c, d^ Different superscript letters indicate the significant statistical difference between (p≤0.05).

Stimulation proliferation (S.I.) index was determined by CFSE dilution and the geometric mean values of the concanavalin A-stimulated duplicates were calculated and divided with the geometric mean values of the PBMC-nonstimulated control duplicates.

**Table S6.** Total **s**pontaneous proliferation and mitogen-induced lymphocyte proliferation in lymphoid organs at 24 hours and 28 days of age.

| **Organ** | **Period** | **Group** | **Immune Cells** | | | | | | |
| --- | --- | --- | --- | --- | --- | --- | --- | --- | --- |
|  |  |  | **T cells (**CD3e^+^) | | **B cells (**CD79a^+^) | | | **Monocyte/Macrophage** | |
|  |  |  | **NS** | **Conc A** | **NS** | | **Conc A** | **NS** | **Conc A** |
| **Spleen** | **24 hours** | **GG** | 13398±1558^ab^ | 15463±1341^ab^ | 5352±1627 | 7610±1407^ab^ | | 12303±1201^b^ | 16538±1447 |
|  |  | **GS** | 11388±875^ab^ | 11674±830^c^ | 4451±1270 | 6779±1568^ab^ | | 13708±2058^ab^ | 16646±2293 |
|  |  | **GMR** | 12505±2866^ab^ | 24252±1995^d^ | 5422±1490 | 9801±1988^ab^ | | 13392±883^b^ | 16438±1102 |
|  |  | **SS** | 16331±1324^a^ | 18838±1738^a^ | 6819±1876 | 7708±1507^ab^ | | 15886±616^a^ | 17265±906 |
|  |  | **SG** | 13026±1374^b^ | 14130±1050^ac^ | 4582±1518 | 13036±2767^a^ | | 13261±2472^ab^ | 17462±1582 |
|  |  | **SMR** | 11425±2448^ab^ | 12558±2040^bc^ | 2882±900 | 5208±832^b^ | | 14890±1497^ab^ | 15775±1773 |
|  |  | *P* - value | 0.2707 | 0.0032 | 0.5511 | 0.2077 | | 0.5253 | 0.9761 |
|  | **28 days** | **GG** | 13531±2773^ab^ | 21361±2068 | 6518±789^c^ | 19794±2642 | | 13190±435 | 14883±267^a^ |
|  |  | **GS** | 9290±1357^b^ | 28972±4877 | 13988±339^b^ | 18578±2198 | | 13568±404 | 15024±872^ab^ |
|  |  | **GMR** | 13361±NA | 15352±NA | 12400±NA | 16727±NA | | 14094±NA | 14052±NA^a^ |
|  |  | **SS** | 19797±2479^a^ | 24062±2020 | 13708±487^b^ | 21016±2203 | | 14282±729 | 15399±1185^ab^ |
|  |  | **SG** | 17252±2929^a^ | 24684±2378 | 15833±1442^ab^ | 20852±2232 | | 13838±537 | 14814±588^ab^ |
|  |  | **SMR** | 14214±1227^a^ | 24858±831 | 16387±122^a^ | 18841±1729 | | 13610±756 | 15604±133^b^ |
|  |  | *P* - value | 0.0701 | 0.4394 | 0.0072 | 0.9123 | | 0.8644 | 0.5633 |
| **Thymus** | **24 hours** | **GG** | 1436±192 | 2393±374 | 1077±159^ab^ | 1504±136^abc^ | | 891.00±206 | 1294±227^a^ |
|  |  | **GS** | 2104±279 | 3464±400 | 1177±81^b^ | 1657±143^ac^ | | 1380±234 | 2233±201^b^ |
|  |  | **GMR** | 1766±347 | 2535±343 | 965.75±79.1^ab^ | 1423±143^ab^ | | 1310±178 | 1551±274^ab^ |
|  |  | **SS** | 1793±134 | 3142±418 | 773.60±43.5^a^ | 1483±129^ab^ | | 1200±268 | 1567±277^ab^ |
|  |  | **SG** | 1325±246 | 3036±493 | 726.80±129^a^ | 1162±27^b^ | | 1161±81 | 1556±98^a^ |
|  |  | **SMR** | 1987±242 | 3061±268 | 1288±102^b^ | 1899±129^c^ | | 1533±176 | 1898±255^ab^ |
|  |  | *P* - value | 0.2412 | 0.3810 | 0.0085 | 0.0122 | | 0.3331 | 0.1148 |
|  | **28 days** | **GG** | 9107±618^b^ | 21230±774 | 7111± 601^a^ | 12622±3164^b^ | | 1605±299 | 2448±437 |
|  |  | **GS** | 15688±2138^a^ | 19271±3092 | 7284±1351^a^ | 15142±2445^ab^ | | 2754±707 | 3581±858 |
|  |  | **GMR** | 20122±NA | 28724±NA | 14815±NA | 22639±NA | | 2155±NA | 3360±NA |
|  |  | **SS** | 18717±1085^a^ | 25208±1437 | 14902±1769^b^ | 23268±4057^a^ | | 3043±797 | 4123±925 |
|  |  | **SG** | 15583±2453^a^ | 20165±2849 | 10699±1076^b^ | 19836±3282^ab^ | | 2951±1003 | 5396±1118 |
|  |  | **SMR** | 16733±1151^a^ | 21025±1322 | 12623±1538^b^ | 16265±1718^ab^ | | 2054±208 | 2717±580 |
|  |  | *P* - value | 0.0251 | 0.2892 | 0.1990 | 0.2690 | | 0.6565 | 0.3235 |
| **mLN** | **24 hours** | **GG** | 4451±1276^b^ | 7615±2299 | 3428±361 | 5265±448^b^ | | 4043±187^ab^ | 4869±235^b^ |
|  |  | **GS** | 5865±968^b^ | 8136±678 | 4124±410 | 5256±335^bc^ | | 3286±231^b^ | 3808±313^c^ |
|  |  | **GMR** | 6610±2280^ab^ | 7765±2478 | 5291±398 | 6342±452^ac^ | | 2097±116^c^ | 2505±189^c^ |
|  |  | **SS** | 10279±1139^a^ | 11443±1423 | 4937±849 | 8975±1486^a^ | | 4987±1103^abc^ | 8125±375^a^ |
|  |  | **SG** | 6491±214^b^ | 7685±367 | 4372±535 | 6006±250^ac^ | | 5177±458^a^ | 6850±745^a^ |
|  |  | **SMR** | 5052±688^b^ | 7389±1667 | 3530±675 | 5644±185^bc^ | | 3007±469^abc^ | 4051±809^bc^ |
|  |  | *P* - value | 0.0749 | 0.4416 | 0.1645 | 0.0522 | | 0.0052 | 0.0004 |
|  | **28 days** | **GG** | 8076±1780 | 9950±1980 | 10702±1269^ab^ | 16197±917 | | 5556±476^abc^ | 6327±318^bd^ |
|  |  | **GS** | 9257±2351 | 10308±1937 | 11261±1155^ab^ | 13782±1502 | | 4265±192^b^ | 4930±455^c^ |
|  |  | **GMR** | 7465±NA | 7925±NA | 5200±NA | 9157±NA | | 1357±NA | 1438±NA |
|  |  | **SS** | 11594±2656 | 13361±3352 | 11338±883^a^ | 15027±664 | | 5665±426^a^ | 21068±1433^a^ |
|  |  | **SG** | 8134±1003 | 9099±1164 | 6524±842^c^ | 12748±271 | | 5222±177^a^ | 5641±243^bc^ |
|  |  | **SMR** | 5629±571 | 8090±744 | 14010±679^b^ | 16554±975 | | 7768±648^c^ | 9156±1232^d^ |
|  |  | *P* - value | 0.4907 | 0.9029 | 0.0164 | 0.0734 | | 0.0094 | 0.0097 |

mLN= mesenteric lymph node

NS = nonstimulated

Com A = concanavalin A

Means in the same column with different superscripts differ significantly (p≤0.05).
